# The burden of respiratory syncytial virus in adults: a systematic review and meta-analysis

**DOI:** 10.1017/S0950268820000400

**Published:** 2020-02-13

**Authors:** M. Tin Tin Htar, M. S. Yerramalla, J. C. Moïsi, D. L. Swerdlow

**Affiliations:** 1 Medical Development, Scientific and Clinical Affairs, Pfizer Inc., Paris, France; 2 Epidemiology of Ageing and Neurodegenerative Diseases, Université de Paris, Institut National de la Santé et de la recherche Médicale (INSERM) U 1153 and Centre of Research in Epidemiology and Statistics (CRESS), Paris, France; 3 Medical Development, Scientific and Clinical Affairs, Pfizer Inc., Collegeville, PA, USA

**Keywords:** Acute respiratory infection, epidemiology, respiratory syncytial virus, surveillance

## Abstract

Respiratory syncytial virus (RSV) is the most common pathogen associated with acute lower respiratory tract infections in young children. RSV is also a major viral pathogen causing severe lung disease in the adult population, particularly among the elderly. We conducted a review of adult RSV studies published from January 1970 to February 2017 to determine the burden of disease among adults worldwide. There were no restrictions on health care setting or definition of RSV infection. A total of 1530 published studies were identified, 95 of which were included in this review. The incidence rates of hospitalised RSV acute respiratory tract infection (ARI) in adults >65 years old ranged from 7.3 to 13.0/10^5^ population in Africa and Asia and from 190 to 254/10^5^ population in the USA. Higher incidence rates (195–1790/10^5^ population) were observed in adults ≥50 years old for outpatient or emergency visits in the USA. Of all ARI patients, RSV accounted for 1–10% in adults and 2–14% in patients with chronic diseases or transplantation. Given the limitations in the existing data, significant efforts should be made to generate evidence on the burden of RSV infections in adults and to estimate the potential impact of future preventive interventions.

## Introduction

Respiratory syncytial virus (RSV) was first recognised as a cause of bronchiolitis among infants in 1957, and is the most commonly identified cause of lower respiratory tract infections (LRTI) in young children [1]. It is an enveloped RNA virus of the *Paramyxoviridae* family and *Pneumovirinae* subfamily [2], displaying minimal antigenic heterogeneity [3]. There are two major subgroups (A and B) with antigenic differences in the P, N, F and G proteins [3]. RSV is transmitted via respiratory tract secretions and survives for more than 24 hours on non-porous surfaces [4]. The incubation period for the infection is 3–5 days, after which infants may develop upper respiratory tract illness, including rhinorrhoea and congestion, with or without fever [4]. Up to 40% of infants progress to LRTI with cough and wheezing, which vary in severity from mild–moderate disease to life-threatening respiratory failure and cyanosis [4]. Nearly all humans are infected with RSV in the early years of life, but the resulting immunity is neither sustained nor complete [4]. RSV infections occur from late fall through early spring in temperate climates over a season of 4–6 months, exhibiting a clear pattern of winter incidence [5]. The seasonality of RSV in tropical and sub-tropical regions is less well defined. In climates with high annual precipitation (e.g. Bangladesh, Guatemala, Thailand), RSV infections usually peak during wet months. In warm/hot climates (e.g. China) and arid (e.g. Egypt) climates, RSV incidence peaks during cooler months [6]. In higher-latitude locations, RSV infection tends to have broader variation, even within individual temperate zones, with peak activity outside of typical winter months [7].

RSV has also been demonstrated to be an important viral pathogen among adults especially those with severe lung disease and the elderly [8]. Adults at higher risk for severe disease include those with underlying cardiopulmonary disease, the severely immunocompromised and frail elderly persons living at home or in long-term care facilities [9]. The burden of RSV may be comparable to that of influenza in the young adult population; for patients aged ≥65 years, RSV has been shown to be second only to influenza among viral pathogens causing cardiopulmonary hospitalisations [10–14]. Both pathogens have similar clinical manifestations and mortality rates [13].

We conducted a systematic review of studies from 1970 to February 2017 describing the incidence and the proportion of RSV in patients with respiratory infections in the adult population. We then pooled the extracted data to determine the proportion of RSV among respiratory infections in the general population across the geographic regions and in populations with various co-morbidities across studies to provide a comprehensive representation of the burden of RSV.

## Methods

A systematic search was conducted for English-language publications within the PubMed database for published papers from 1 January 1970 to 15 February 2017. We selected 1970 as the starting point, as this was the approximate time when RSV was becoming recognised as a potentially serious pathogen among adults [15–17]. The search strings included terms related to RSV (‘respiratory syncytial virus’, ‘respiratory syncytial viruses’, ‘RSV’, ‘respiratory syncytial virus infection’), outcomes of interest (‘incidence’, ‘mortality’, ‘prevalence’, ‘risk factor’, ‘risk’, ‘distribution’, ‘etiology’, ‘aetiology’, ‘epidemiology’) and study design (‘surveillance’, ‘observational’, ‘case-control’).

Included in this analysis were original observational studies involving adults aged ≥18 years and reporting RSV infection incidence, the proportion of RSV among individuals with acute respiratory infections (ARI), and/or incidence and proportion of RSV among those with underlying high-risk conditions. There was no restriction on the healthcare settings or the definition of RSV infection that was used in the included studies. Studies were excluded if the sample size of the entire study population was less than 50 persons, if the definition of RSV-related illness was unclear, or if RSV was investigated only as a co-infection. Case reports, narrative reviews, commentaries, modelling and review articles were also excluded.

Incidence rate data in the general population were collected independently as each study described. The attack rate was collected or calculated as the cumulative incidence rate of RSV among all patients followed in a particular cohort for a defined time period. The proportion of RSV was calculated as the proportion of RSV-confirmed cases among the total cases of respiratory illness studied, which could be ARI, influenza-like illness (ILI), severe acute respiratory infection (SARI), respiratory viral infection (RVI) or pneumonia.

**Fig. 1.**
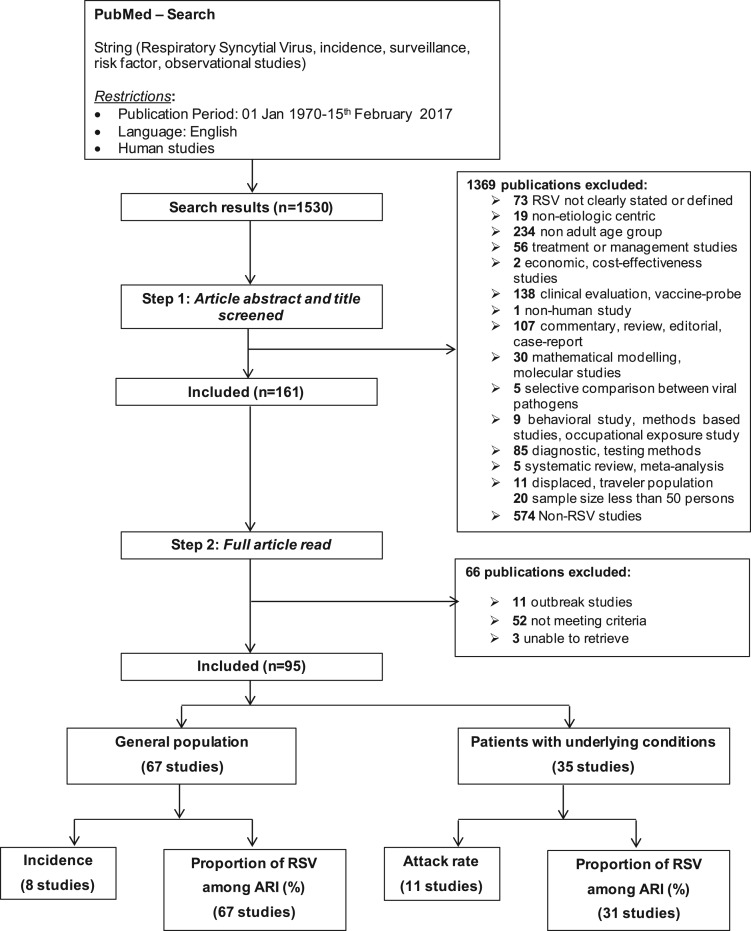
Flow chart of the selection process of articles. *Note that some studies included more than one outcome.

Articles were screened according to the Preferred Reporting Items for Systematic Reviews and Meta-Analyses (PRISMA) model [18], as shown in the flow chart in Figure 1. The meta-analysis was conducted on the pooled data using the Metaprop package in Stata 12 software [19]. The pooled proportion rates were estimated by using the Freeman–Tukey double arcsine transformation method (PFT), which allowed for studies with estimated proportions close to 0 or 1 to be included [19, 20]. A random-effects meta-analysis was performed to allow for heterogeneity across studies [21]. Heterogeneity was assessed using the *χ*^2^-based *Q* test [22] and *I*^2^ statistic [23]. Stratified analyses for the detection of potential sources of heterogeneity and meta-regression for the effect of an individual study on the overall outcomes were conducted only when there were at least 10 studies included in the meta-analysis. In order to compensate for the lower power of the test of heterogeneity, a *P*-value of <0.1 was considered statistically significant [21]. All statistical analyses were conducted in Stata 12 software.

## Results

A total of 1530 records were identified from the initial database search. After screening titles and abstracts for exclusions, 161 papers were included for full-text review. Of those, 95 articles met the predefined eligibility criteria and were included in this review: 67 studies provided RSV information for the general population and 35 included patients with underlying risk conditions.

### Incidence of RSV in the general population

Eight studies provided estimates of RSV incidence. All were prospective surveillance studies conducted in the USA, Thailand, Egypt or Kenya between 2006 and 2012 [24–29]. Five studies described the incidence rates of hospitalised RSV-related ARI ranging from 0.9 to 4.1/10^5^ in adults 20–49 years old and from 7.3 to 13.0/10^5^ in adults over 65 years old in Thailand and Africa. Substantially higher rates were observed in the USA with a range of 128–340/10^5^ population for emergency department settings. Within the same studies, adults over 65 years old showed higher incidence rates compared to those in younger age groups. Six studies described the incidence rate of RSV in out-patient clinic settings: three studies from Kenya, one from Egypt and two from the USA. The incidence rate of RSV-related ILI was close to zero for those over 50 years old in Egypt and 0–10/10^5^ for adults in Kenya (2007–2010); the incidence rate of RSV-ARI was 195–1990/10^5^ in those over 50 years old in the USA (2006–2010) (Table 1).

**Table 1.** Incidence estimates for RSV by setting and country

Country	Setting	Outcomes	Population	Incidence (/100 000 population)	Reference
Hospitalisation					
Egypt 2009–2012	Hospital (rural)	ARI	20–49 years	4.1 (2–4)	Rowlinson *et al*. [24]
			≥50 years	5.2 (0.3–10)	
			50–64 years	5.9 (0.3–12)	
			≥65 years	NA	
	Hospital (urban)	ARI	20–49 years	1.3 (0–0.2)	Rowlinson *et al*. [24]
			≥50 years	6.7 (3–11)	
			50–64 years	6.6 (3–11)	
			≥65 years	7.3 (0.4–21)	
Kenya 2009–2012	Hospital	SARI	≥5 years	10 (0–20)	Emukule [25]
Thailand 2008–2011	Hospital	ALRI	20–49 years	0.9 (0.6–1.1)	Naorat [26]
			50–64 years	4.0 (3.2–4.9)	
			≥65 years	13.0 (10.8–15.2)	
	Hospital	Pneumonia chest X-ray confirmed	20–49 years	0.2 (0.1–0.3)	Naorat [26]
			50–64 years	1.3 (0.9–1.8)	
			≥65 years	3.1 (2.1–4.0)	
USA 2006–2009	Hospital	ARI	50–64 years	82 (33–123)	Widmer *et al*. [30]
			≥65 years	254 (131–380)	
			≥50 years	150 (86–198)	
USA 2006–2010	Hospital	ARI	18–49 years	21 (10–42)	Widmer *et al*. [31]
			50–64 years	67 (33–134)	
			≥65 years	190 (104–340)	
			≥50 years	112 (71–177)	
			≥18 years	55 (37–81)	
Outpatients/emergency visits					
Kenya 2007–2011	Clinics (slum)	SARI	≥18 years	440	Bigogo *et al*. [27]
		ILI	≥18 years	0	Bigogo *et al*. [27]
	Clinics (rural)	SARI	≥18 years	80	Bigogo *et al*. [27]
		ILI	≥18 years	10	Bigogo *et al*. [27]
Kenya 2009–2012	Clinics	ILI	≥5 years	90 (40–220)	Emukule *et al*. [25]
Kenya 2007–2010	Clinics	ARI	≥5 years	440 (350–530)	Feikin *et al*. [29]
Egypt 2009–2012	Clinics (rural)	ILI	20–49 years	257 (32–1163)	Rowlinson *et al*. [24]
			≥50 years	0 (0–1303)	
	Clinics (urban)	ILI	20–49 years	939 (9–7114)	
			≥50 years	0 (0–13 841)	
USA					McClure *et al*. [28]
2006–2007	Clinics, ED	ARI	≥50 years	1100 (750–1610)	
2007–2008				1790 (1320–2440)	
2008–2009				1660 (1250–2210)	
2009–2010				1590 (1220–2080)	
USA 2006–2010	Clinics, ED	ARI	50–59 years	1240 (990–1560)	McClure *et al*. [28]
			60–69 years	1470 (1100–1960)	
			≥70 years	1990 (1530–2580)	
			≥50 years	1540 (1320–1800)	
USA 2006–2010	ED	ARI	18–49 years	132 (67, 253)	Widmer *et al*., 2014 [31]
			50–64 years	128 (44, 354)	
			≥65 years	340 (117, 908)	
			≥50 years	195 (90, 408)	
			≥18 years	154 (93, 254)	

ALRI, acute lower respiratory infection; ARI, acute respiratory infection; CXR, chest x-ray; ED, emergency department; ILI, influenza-like illness; SARI, severe acute respiratory infection.

### Proportion of RSV among respiratory infections in the general population

A total of 67 studies contributed 140 estimates to the proportion of RSV in ARI in the general population. Study characteristics have been summarised by continent or major region (Australia was excluded, as there was no publication that estimated RSV in adults in that country/continent (Appendix Table 2)).

#### Africa

**Fig. 2.**
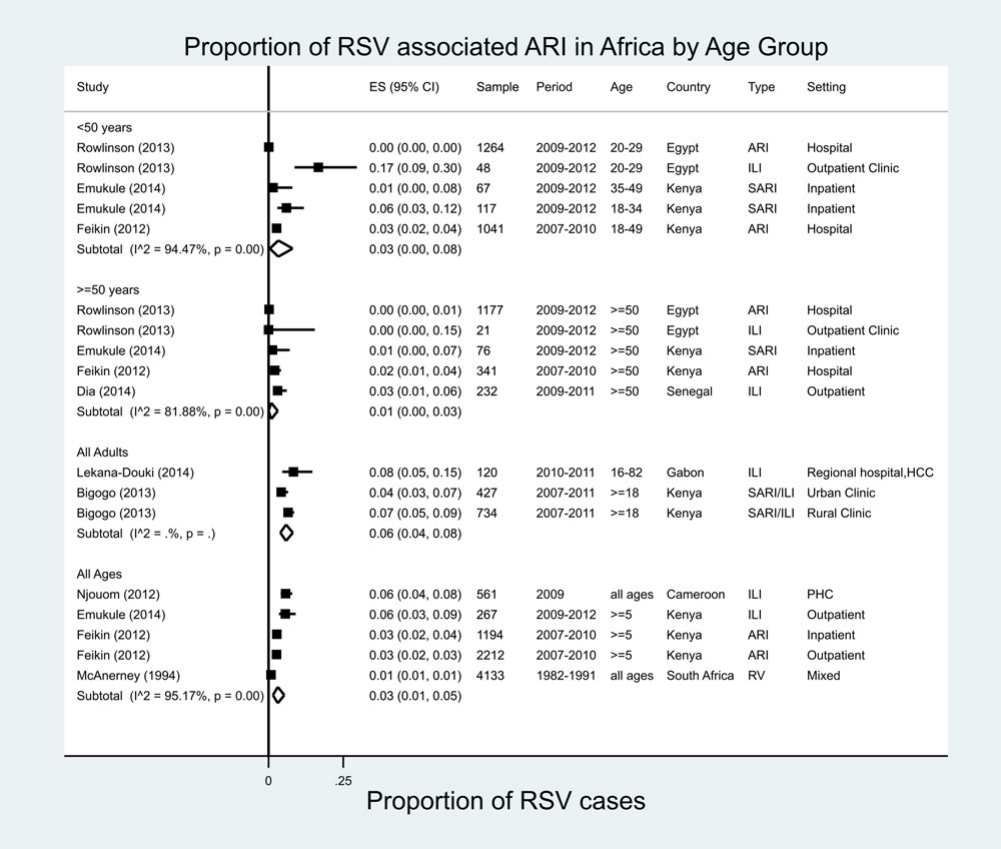
Proportion of RSV-associated ARI/ILI in the Africa region.

There were 18 estimates from eight studies covering six countries in Africa (Fig. 2) [24, 25, 27, 29, 32–35]; 13 of 18 estimates were for adults aged ≥18 years. In individuals aged ≥50 years, RSV was found in proportions ranging from 0% in Egypt (ARI, 2009–2012) to 3% in Senegal (ILI, 2009–2011). The meta-analysed proportion of RSV was 1% (95% CI 0–3%) with marked heterogeneity across studies in terms of study populations (*I*^2^ = 81.9%).

#### Central America/Caribbean

**Fig. 3.**
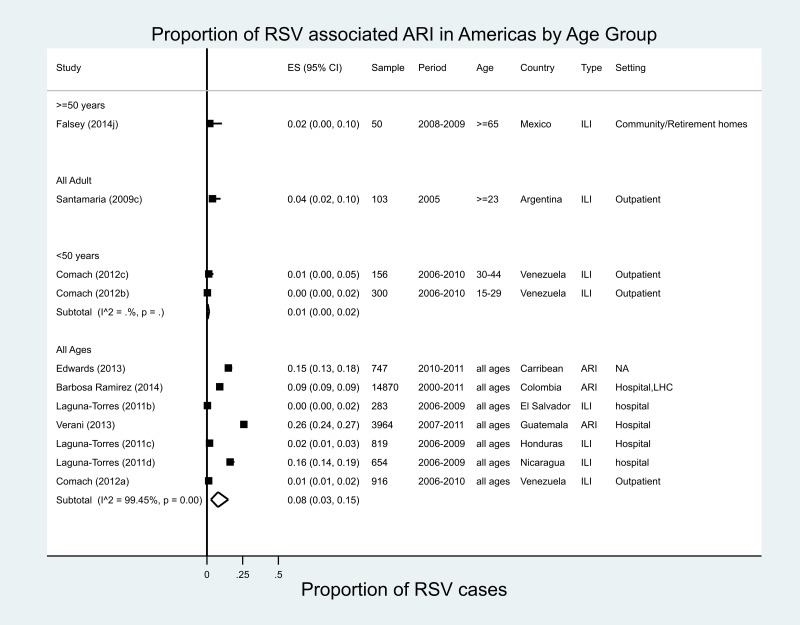
Proportion of RSV-associated ARI/ILI in the Americas region.

A total of 11 estimates were reported for the proportion of RSV from seven studies in eight countries of Central America and the Caribbean [36–42], ranging from 0% in El Salvador (ILI, 2006–2009) to 26% in Guatemala (ARI, 2007–2011) for all ages. One study was conducted in 24 Caribbean countries from 2010 to 2011, the year immediately following the influenza pandemic of 2009–2010 (Fig. 3) [42], and found that RSV accounted for 15% of all ARI. Our meta-analysis estimated an RSV proportion of 8% (95% CI 3–5%) of ARI/ILI in all age groups for Central America/Caribbean studies, with very high heterogeneity (*I*^2^ = 99.5%). Only one study in Mexico provided data in patients ≥65 years of age, in whom RSV accounted for 2% of all moderate-to-severe ILI [36].

#### Asia

**Fig. 4.**
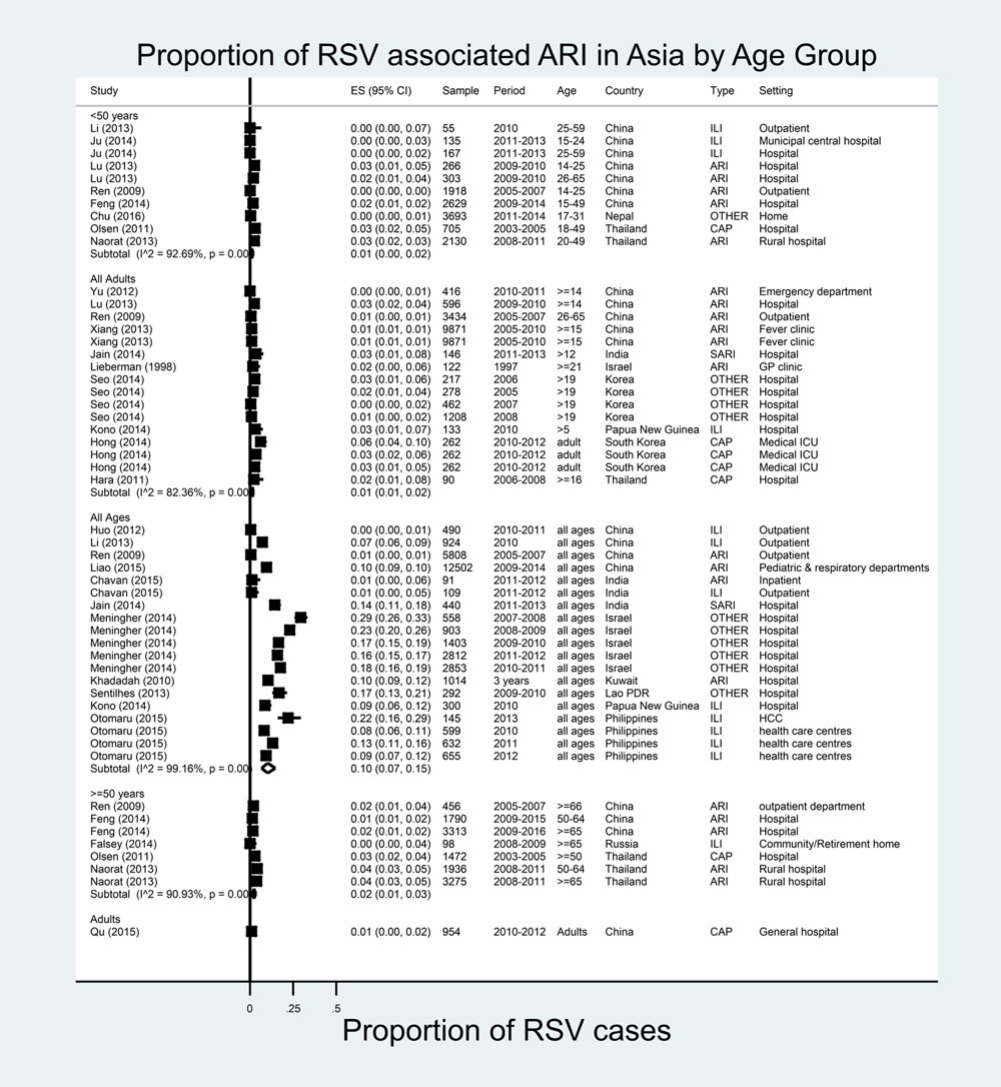
Proportion of RSV among ARI/ILI in adults in Asia.

A total of 53 estimates were available from 25 studies covering 11 Asian countries (10 studies were from China; three from Thailand; two each from South Korea, India and Israel; and one each from Nepal, Laos, Papua New Guinea, Philippines, Kuwait and Russia) (Fig. 4) [26, 36, 43–65]. China alone contributed to 22 estimates for the period of 2005–2014. Across all age groups, RSV accounted for a proportion of ILI ranging from 0.4% in Nanjing, China (2010–2011) to 29.4% in Israel (2007–2008). In the meta-analysis, the proportion of ILI cases attributable to RSV was 10% (95% CI 7–15%) with very high heterogeneity (*I*^2^ = 99.2%). The highest proportion was observed in Israel (16–29% in all ages for the periods 2007–2012) [62]. Only seven estimates were for adults ≥50 years of age; RSV accounted for the proportions of ARI ranging from 1.7% in China (2009–2015) to 3.8% in Thailand (2008–2011) in this age group; in the meta-analysis, the proportion was 2% (95% CI 1–3%) with high heterogeneity (*I*^2^ = 90.9%). In adults ≥65 years of age in Taiwan during 2008–2009, the proportion of RSV was almost zero in moderate-to-severe ARI [36]. The heterogeneity remained high for all sub-categories except for community-acquired pneumonia within illness definition (Appendix Table 3). Meta-regression analyses showed that none of the sub-categories were significantly associated with heterogeneity (Appendix Table 4).

#### Europe

**Fig. 5.**
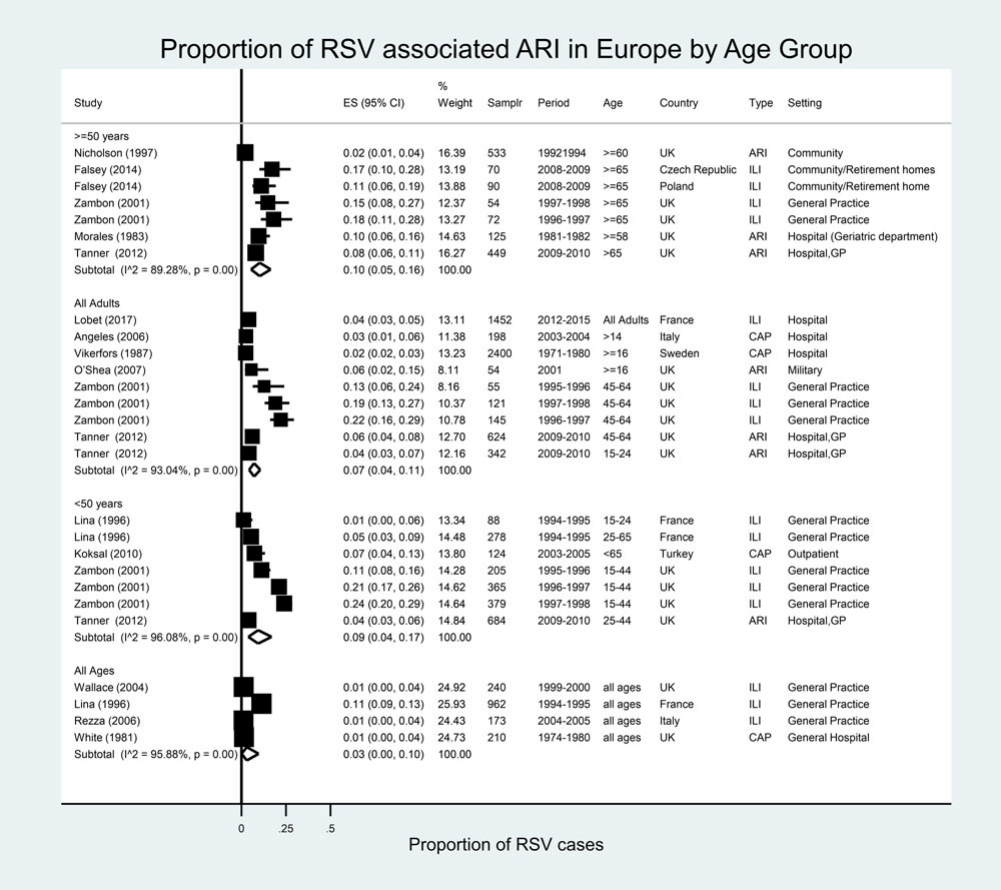
Proportion of RSV among ARI/ILI in adults in Europe.

In Europe, 27 estimates from 14 studies were available for eight countries (Fig. 5) [36, 66–77]. RSV accounted for 1% (Italy, 2004–2005 and UK, 2009–2010) to 11% (France, 1994–1995) of all ILI in patients of all ages. In adults ≥50 years of age, RSV accounted for 2% (UK, 1992–1994) to 18% (UK, 1996–1997) of ILI. The proportion of ARI/ILI or CAP cases attributable to RSV in the meta-analysis was 10% (95% CI 5–16%), with high heterogeneity (*I*^2^ = 89.3%).

#### North America

**Fig. 6.**
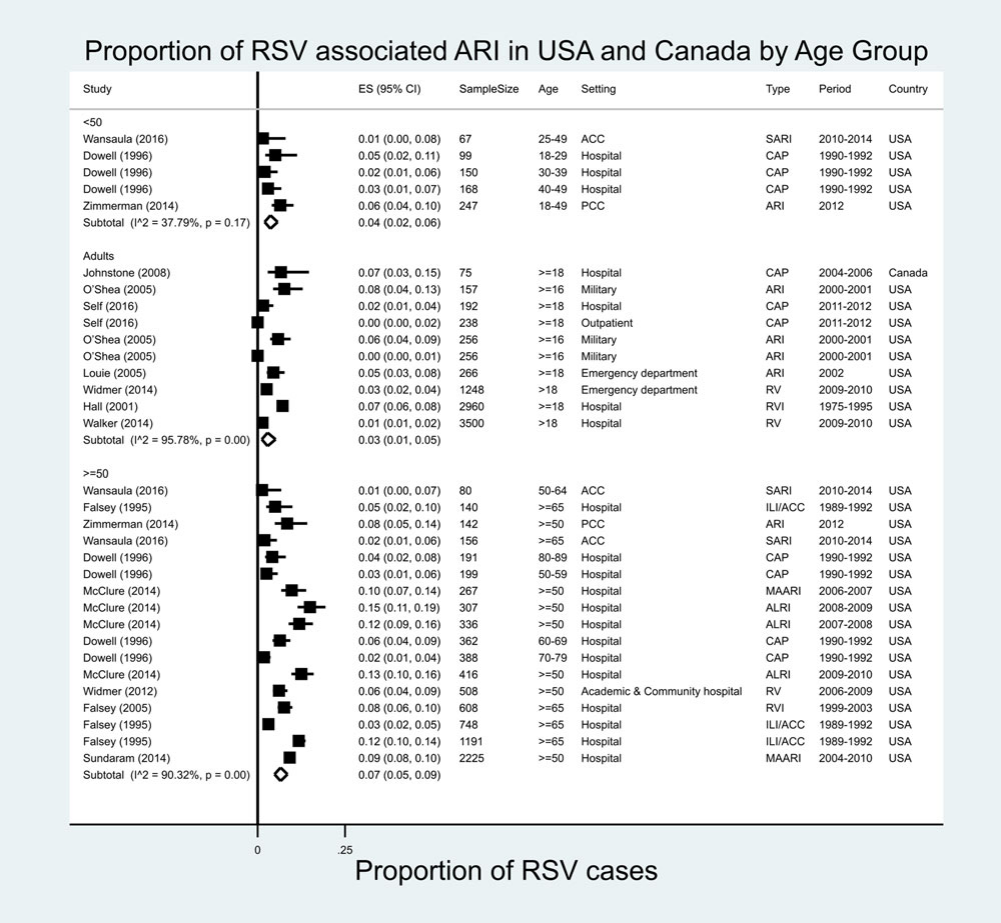
Proportion of RSV among ARI/ILI in adults in the USA and Canada.

In North America, 32 estimates were available from 15 studies in the USA and one from Canada (Fig. 6) [10, 13, 14, 28, 30, 31, 78–86]. In adults ≥18 years of age in the USA, RSV accounted for varying proportions of all ARI cases, ranging from 1.4% in Chicago (2009–2010) to 8% (adults, military, 2000–2001). Within the meta-analysis population, the proportion of ARI/ILI or CAP cases attributable to RSV in North America was 3% (95% CI 1–5%), with very high heterogeneity (*I*^2^ = 95.8%). In adults ≥50 years of age, the proportion of ARI cases attributable to RSV ranged from 1.3% in southern Arizona (50–64 years, 2010–2014) to 15% in Wisconsin (≥65 years, 2008–2009). The proportion of RSV in the meta-analysis population among those over 50 years of age with ARI/ILI or CAP was 7% (95% CI 5–9%), with high heterogeneity between studies (*I*^2^ = 90.3%). The meta-regression analyses showed that the age group variable was the only source of heterogeneity identified across the studies (Appendix Table 5).

### Incidence of RSV in patients with underlying diseases

A total of 43 studies were included for the analysis in patients with underlying diseases; 11 studies provided 16 estimates of RSV infection attack rates in different cohorts, mostly in patients with organ or stem cell transplantation [85, 87–96]. The RSV attack rate varied widely across cohorts, ranging from 2.1% (a cohort of Hematopoietic Stem Cell Transplantation (HSCT) patients followed from 1997 to 1998 in Europe) to 19.6% (a cohort of adult HSCT patients followed from 1992 to 1993 in the USA) (Table 2), with the exception of 30% and 43% found in one study among US adult patients with multiple myeloma with autologous HSCT and with chemotherapy, respectively. In transplant patients, a higher rate of RSV infection was observed in patients undergoing autologous stem cell transplantation compared to those undergoing chemotherapy during the follow-up year 1997–1998 [92]. Two studies in the USA described the RSV attack rates in different cohorts either healthy elderly (≥65 years old) or with chronic heart failure (CHF) or chronic pulmonary diseases (CPD) [85, 97]. While RSV attack rates varied according to years, the higher rates were generally observed in the cohort with CHF or CPD already admitted to a hospital for ARI (7.7–13.2%) compared to those of healthy elderly (2.8–7.1%) and with CHF and CPD (3.6–9.7%) (Table 2).

**Table 2.** Incidence (attack rate) of RSV infection in patients with underlying conditions

Conditions (countries)	Cumulative attack rate	Cohort	Study population	Study period	Reference
HSCT (Europe)	2.1%	1973	All ages	1997–1998	Ljungman *et al*. [87]
Allogeneic HSCT (Spain)	7.5%	172	Adults	1999–2003	Martino *et al*. [88]
Autologous HSCT (Spain)	2.9%	240	Adults	1999–2003	Martino *et al*. [88]
Allogeneic HSCT (Sweden)	11.6%	275	All ages	2000–2007	Avetisyan *et al*. [89]
Allogeneic + autologous HSCT (USA)	19.6%	102	Adults	1992–1993	Whimbey *et al*. [90]
Allogeneic + autologous HSCT (USA)	10.7%	112	Adults	1993–1994	Whimbey *et al*. [90]
Allogeneic + autologous HSCT (USA)	15.4%	214	Adults	1992–1994	Whimbey *et al*. [90]
Allogeneic HSCT (USA)	8.8%	548	All ages	1994–1999	Small *et al*. [91]
Allogeneic HSCT (USA)	5.3%	394	Adults	1994–1999	Small *et al*. [91]
Multiple myeloma (USA)	38.1%	147	Adults	1997–1998	Anaissie *et al*. [92]
Multiple myeloma with autologous HSCT (USA)	43.3%	90	Adults	1997–1998	Anaissie *et al*. [92]
Multiple myeloma with chemotherapy (USA)	29.8%	57	Adults	1997–1998	Anaissie *et al*. [92]
HSCT (USA)	5.8%	122	All ages	2000–2004	Peck *et al*. [93]
Lung transplant (USA)	4.1%	122	Adults	1992–1997	Palmer *et al*. [94]
CHF or COPD (USA)	7.5%	107	Adults	1996–1998	Walsh *et al*. [97]
CHF or COPD (USA)	9.7%	206	Adults	1999–2000	Falsey *et al*. [85]
CHF or COPD (USA)	6.6%	271	Adults	2000–2001	Falsey *et al*. [85]
CHF or COPD (USA)	3.6%	195	Adults	2001–2002	Falsey *et al*. [85]
CHF or COPD (USA)	5.2%	210	Adults	2002–2003	Falsey *et al*. [85]
CHF or COPD (USA)	10.4%	540	Adults	1999–2003	Falsey *et al*. [85]
Healthy elderly (USA)^a^	5.7%	212	≥65 years	1999–2000	Falsey *et al*. [85]
Healthy elderly (USA)^a^	7.1%	270	≥65 years	2000–2001	Falsey *et al*. [85]
Healthy elderly (USA)^a^	2.8%	180	≥65 years	2001–2002	Falsey *et al*. [85]
Healthy elderly (USA)^a^	3.1%	295	≥65 years	2002–2003	Falsey *et al*. [85]
Healthy elderly (USA)^a^	7.6%	608	≥65 years	1999–2003	Falsey *et al*. [85]
CHF or COPD patients admitted with ARI (USA)	7.7%	274	≥65 years	1999–2000	Falsey *et al*. [85]
CHF or COPD patients admitted with ARI (USA)	13.2%	296	≥65 years	2000–2001	Falsey *et al*. [85]
CHF or COPD patients admitted with ARI (USA)	11.4%	434	≥65 years	2001–2002	Falsey *et al*. [85]
CHF or COPD patients admitted with ARI (USA)	9.6%	384	≥65 years	2002–2003	Falsey *et al*. [85]
CHF or COPD patients admitted with ARI (USA)	10.2%	1388	≥65 years	1999–2003	Falsey *et al*. [85]
Multiple myeloma (Australia)	4.5%	330	Adults	2009–2012	Teh *et al*. [95]

HSCT, hematopoietic stem cell transplantation.

a Healthy elderly where 16% of the cohort living with any lung or heart disease and 10% with diabetes mellitus.

**Table 3.** Proportion with RSV among patients with respiratory infections and underlying conditions

Conditions	Proportion of RSV	Cases with outcomes	Outcomes	Age	Period	Reference
Chronic cardio-pulmonary diseases						
Asthma (Australia)	1.3%	79 cases	AE-asthma	Adults	1993–1994	Teichtahl *et al*. [98]
Asthma (UK)	1.2%	84 episodes	AE-asthma	Adults	1990–1992	Nicholson *et al*. [99]
COPD (Australia)	0.6%	148 episodes	AE-COPD	>50 years	2003–2005	Hutchinson *et al*. [100]
COPD (Canada)	7.4%	108 cases	AE-COPD	>50 years	2002–2003	De Serres *et al*. [101]
COPD (France)	4.9%	122 cases	ARF/ACF	>60 years	2002–2004	Carrat *et al*. [102]
COPD (Greece)	40.5%	200 cases	AE-COPD	>18 years	2008–2009	Dimopoulos *et al*. [103]
COPD (Hong Kong)	2.4%	245 cases	AE-COPD	>60 years	2004–2005	Ko *et al*. [104]
COPD (Iran)	7.6%	170 cases	AE-COPD	66 ± 8.9 years	2010–2012	Hosseini *et al*. [105]
COPD (Iran)	6.3%	96 cases	Stable COPD	63 ± 9.1 years	2010–2012	Hosseini *et al*. [105]
COPD (Switzerland)	3.5%	86 cases	sAE-COPD	>60 years	2007–2008	Kherad *et al*. [106]
COPD (UK)	14.2%	120 cases	AE-COPD	66.6 ± 7.1	16 months	Seemungal *et al*., 2001 [107]
COPD (UK)	23.5%	68 cases	COPD	67.7 ± 8.1	16 months	Seemungal *et al*. [107]
COPD (USA)	8.0%	50 cases	AE-COPD	Adults	2002–2003	Martinello *et al*. [108]
COPD (USA)	7.9%	76 cases	msAE-COPD	≥50 years	2003–2004	Camargo *et al*. [109]
COPD/CHF (USA)^a^	8.5%	59 illness	ARI	Adults	1996–1997	Walsh *et al*. [97]
COPD/CHF (USA)^a^	3.1%	96 illness	ARI	Adults	1996–1998	Walsh *et al*. [97]
COPD (USA)	6.5%	138 cases	ARI	≥50 years	2001	Sundaram *et al*. [84]
CHF (USA)	6.5%	124 cases	ARI	≥50 years	2001	Sundaram *et al*. [84]
Chronic heart/lung disease (USA)	13.7%	146 illness	ARI	≥65 years	1999–2000	Falsey *et al*. [85]
Chronic heart/lung disease (USA)	11.3%	160 illness	ARI	≥65 years	2000–2001	Falsey *et al*. [85]
Chronic heart/lung disease (USA)	6.1%	115 illness	ARI	≥65 years	2001–2002	Falsey *et al*. [85]
Chronic heart/lung disease (USA)	10.7%	103 illness	ARI	≥65 years	2002–2003	Falsey *et al*. [85]
Hospitalised chronic heart/lung disease (USA)	7.3%	289 illness	ARI	≥65 years	1999–2000	Falsey *et al*. [85]
Hospitalised chronic heart/lung disease (USA)	12.7%	307 illness	ARI	≥65 years	2000–2001	Falsey *et al*. [85]
Hospitalised chronic heart/lung disease (USA)	9.7%	465 illness	ARI	≥65 years	2001–2002	Falsey *et al*. [85]
Hospitalised chronic heart/lung disease (USA)	9.0%	410 illness	ARI	≥65 years	2002–2003	Falsey *et al*. [85]
Respiratory disease (USA)	3.1%	671 cases	ARI	Adults	2009–2010	Widmer *et al*. [31]
Cardiovascular disease(USA)	3.3%	427 cases	ARI	Adults	2009–2010	Widmer *et al*. [31]
Chronic lung disease	2.0%	99 cases	SARI	All ages	2000–2014	Wansaula *et al*. [78]
Chronic cardiac disease	0%	80 cases	SARI	All ages	2000–2014	Wansaula *et al*. [78]
Lung disease (USA)	10.8%	167 cases	RVI	Adults	2009–2010	Walker *et al*. [83]
Cardiovascular disease (USA)	14.0%	107 cases	RVI	Adults	2009–2010	Walker *et al*. [83]
Transplant patients						
HSCT (Europe)	2.1%	1973 cases	ARI	All ages	1997–1998	Ljungman *et al*. [87]
Allogenic HSCT (Spain)	23.5%	51 cases	ARI	Adults	1999–2003	Martino *et al*. [88]
Autologous HSCT (Spain)	12.5%	32 cases	ARI	Adults	1999–2003	Martino *et al*. [88]
HSCT (USA)	54.1%	37 cases	ARI, RV+	Adults	1992–1993	Whimbey *et al*. [90]
HSCT (USA)	43.3%	30 cases	ARI, RV+	Adults	1993–1994	Whimbey *et al*. [90]
HSCT (USA)	49.3%	67 cases	ARI, RV+	Adults	1992–1994	Whimbey *et al*. [90]
HSCT (USA)	20.0%	30 cases	RVI	All ages	2000–2004	Peck *et al*. [93]
HSCT (USA)	41.4%	82 cases	RVI	Paediatrics	1993–2006	Lo *et al*. [110]
SOT (USA)	43.6%	78 cases	RVI	Paediatrics	1993–2006	Lo *et al*. [110]
Immunocompromised patients						
Immunodeficiency (USA)	2.8%	680 cases	ARI	≥18 years	2009–2010	Widmer *et al*. [31]
HIV+ (Kenya)	9.5%	179 cases	ARI	≥5 years	2007–2010	Feikin *et al*. [29]
Leukaemia (USA)	10.3%	87 cases	ARI	All ages	1993–1994	Whimbey *et al*. [111]
Immunocompromised (USA)	8.6%	222 cases	RVI	Adults	2009–2010	Walker *et al*. [83]
Hematologic malignancy (USA)	13.3%	83 cases	RVI	Adults	2009–2010	Walker *et al*. [83]
Chemotherapy last 30 days (USA)	15.0%	60 cases	RVI	Adults	2009–2010	Walker *et al*. [83]
Chemotherapy (USA)	41.7%	48 cases	RVI	Paediatrics	1993–2006	Lo *et al*. [110]
Multiple myeloma (Australia)	20.0%	75 cases	RVI	Adults	2009–2012	Teh *et al*. [95]
Other chronic diseases						
Diabetes mellitus (USA)	2.3%	302 cases	ARI	Adults	2009–2010	Widmer *et al*. [31]
Diabetes mellitus (USA)	13.2%	114 cases	RVI	Adults	2009–2010	Walker *et al*. [83]
Liver disease (USA)	3.7%	27 cases	ARI	≥50 years	2001	Sundaram *et al*. [84]
Renal disease (USA)	11.1%	162 cases	ARI	≥50 years	2001	Sundaram *et al*. [84]
Renal disease (USA)	15.6%	96 cases	RVI	Adults	2009–2010	Walker *et al*. [83]
Metabolic disorder (Arizona, USA)	1.8%	109 cases	SARI	All ages	2000–2014	Wansaula *et al*. [78]
Hypertension (Arizona, USA)	0.7%	136 cases	SARI	All ages	2000–2014	Wansaula *et al*. [78]
Chronic illness (USA)	2.8%	1013 cases	ARI	≥18 years	2009–2010	Widmer *et al*. [31]
Nursing home (USA)	27.5%	149 illness	ARI	Adults	1989–1990	Falsey *et al*. [112]
Daycare centres elderly (USA)	9.7%	165 illness	ARI	Elderly	1992–1993	Falsey *et al*. [12]
Geriatric long-stay wards (USA)	7.5%	159 cases	ARI	Elderly	1981–1982	Morales *et al*. [68]
Allergy (San Francisco, USA)	6.7%	75 cases	ARI	Adults	2002	Louie *et al*. [82]
Tobacco smoke exposure^b^ (USA)	1.9%	579 cases	ARI	≥18 years	2009–2010	Widmer *et al*. [31]
Mixed conditions (Sweden)	2.4%	127 cases	CAP	Adults	NA	Berntsson *et al*. [113]

ACF, acute cardiac failure; AE, acute exacerbation; ARF, acute respiratory failure; ARI, acute respiratory tract infection; CAP, community-acquired pneumonia; CHF, congestive heart failure; COPD, chronic obstructive pulmonary disease; ES, estimates; HIV, human immunodeficiency virus; msAE, moderate-to-severe acute exacerbation; RV+, respiratory virus positive; RVI, respiratory virus infection; sAE, severe acute exacerbation; SARI, severe acute respiratory infection. SOT, Solid Organ Transplant.

a Class III or IV CHF by New York State Heart Association.

b Smoked or had significant environmental tobacco exposure within the last 6 months.

### Proportion of RSV among respiratory infections in patients with underlying diseases

A total of 38 studies provided 67 estimates for the proportion of RSV among cases of ARI/ILI in patients with underlying diseases; 17 studies included 32 estimates in patients with chronic respiratory and/or cardiac diseases [31, 78, 83–85, 97–109] (Appendix Table 5). In patients with chronic obstructive pulmonary disease (COPD) or asthma, RSV was responsible for 0.6–8.0% of acute exacerbation of COPD (AE-COPD) across most studies. However, some markedly higher proportions were described both in a 2-year prospective, descriptive study in a tertiary care hospital in Greece in 2008–2009 (40.5%) [103], and in a prospective cohort study in the UK (14.2%) [107]. Two prospective studies compared the rates of RSV detection in patients with AE-COPD *vs.* patients with stable COPD to assess the association between viral infections and acute exacerbations in COPD patients [105, 107]. The case–control study in Iran showed a comparable rate of RSV (7.6%) in patients with stable disease *vs.* patients with AE-COPD (6.3%), while the prospective cohort study in the UK showed a higher rate of RSV in patients with AE-COPD episodes (23.5%) *vs.* patients with stable COPD (14.2%). The statistical significance of these comparisons was not assessed. In patients with chronic respiratory or cardiovascular diseases, the proportion of RSV among ARI cases ranged from 0% to 13.3%. In the USA, a prospective surveillance study in adults with the substantial cardiopulmonary disease described higher RSV prevalence compared to those seen in other studies, with a range of 6.1–13.7% during the years 1999–2003 [85].

Among transplant patients in Europe, RSV accounted for 12.5–50% of RVI cases and 2.1% of respiratory tract infection [87, 88, 90, 93, 110]. In immunocompromised patients, RSV accounted for 2.8–10.3% of all ARI cases, and 8.6–20.0% of all RVI cases [29, 31, 83, 95, 110, 111]. Studies of a variety of chronic diseases described a wide range of proportions of ARI or RVI attributable to RSV [12, 31, 68, 78, 82–84, 112, 113]. The highest rate was reported in a study of nursing home patients in the USA, revealing that 27.5% of ARI were due to RSV from 1989 to 1990 [112].

## Discussion

This review describes the incidence and the proportion of RSV among patients with respiratory infections in adult populations worldwide. We identified and included relevant studies published since 1970, from all regions of the world and including different high-risk groups to provide a comprehensive picture of the RSV burden.

RSV is the most common pathogen identified in young children with acute lower respiratory infections (ALRI), primarily pneumonia and bronchiolitis [114]. In our review, the incidence of ILI/ARI due to RSV was generally lower in adults compared to that in young children. In addition, the incidence rates of RSV-related ARI among hospitalised subjects were 0.9–4.1 and 7.3–13.0/100 000 population in adults 20–49 and >65 years old, respectively, in Egypt and Kenya during 2009–2012. These rates were very low compared to those recently published in the USA and globally. In a recent prospective study in the USA, the overall seasonal incidence of medically-attended RSV illness in ≥60 years of age was 139/10 000 during 2006–2016 despite the decreasing temporal trend since 2011–2012 [115]. A recent review of RSV hospitalisation rates in adults ≥65 years of age estimated them to be 1/1000 and 0.3/1000 person-years in industrialised and developing countries, respectively, in 2015 [116]. Nevertheless, the incidence rates varied widely across countries and study periods. Population-based studies evaluating incidence rates in adults were very few and used highly variable methods, so drawing inferences from our results is challenging. While the incidence of RSV in adults is substantially lower than that observed in young children (20 and 27/1000 infants <6 months old in developing and industrialised countries, respectively, in 2015 [114]), the total number of RSV-related hospitalisations could be much greater for the adult population compared to young children. For example, it is estimated that RSV causes an average of 177 000 hospitalisations and 14 000 deaths annually in adults >65 years compared to 52 527 hospitalisations in children <5 years old in the USA [117].

In our review, RSV was responsible for 1–7% of ILI-ARI in adults, and 1–10% of ILI-ARI in adults ≥50 years old. These reported proportions of RSV were higher in Europe and the USA compared to lower-income countries (10% and 7% in Europe and USA, respectively, and 1–2% in Africa and Asia), but these differences could be due to methodological differences in study designs, health care settings, health care-seeking behaviours, health care access in general and diagnostic facilities, as well as true epidemiological differences in disease risk.

Older adults and people living with underlying diseases are known to be at a higher risk of respiratory infections, RSV and influenza in particular, compared to healthy young adults. In some studies, RSV infections occurred more frequently than influenza infections and may result in greater morbidity and mortality in transplant and immunocompromised patients, and in patients with chronic respiratory and congestive heart diseases [118, 119]. In our review, about 2–20% of HSCT patients suffered from at least one RSV infection during 1–5 years post transplantation in different prospective cohort studies [87–91]. Similarly, about 8–13% of patients with chronic lung or heart diseases suffered from RSV illness during 1–3 years of follow-up in 1996–2003 [85]. A recent cohort study in nine northern hemisphere countries described the same rate of 13% in 330 patients with chronic heart and lung diseases followed from 2011–2012 through 2014 [120]. These attack rates were substantially higher than those observed in the healthy adults [84, 118].

There are several reasons why our analysis for adult populations is likely to have substantially underestimated RSV disease burden. First, case detection in many studies was based on testing for RSV in patients with clinical syndromes such as ILI, ARI or SARI. A majority of RSV cases in adults may not be captured in these studies because RSV in older children and adults is often mild and afebrile, occurs with non-specific symptoms and lasts for less than a week [115]. Second, the use of different diagnostic methods at different time points in the clinical course of illness may have a large impact on test results. Rapid antigen tests are known to have poor sensitivity in older adults and are not optimal for the detection of RSV [121, 122]. Third, several studies, especially in Europe, were based on influenza surveillance platforms which may not be the most appropriate for estimating the RSV burden in adults, as the seasonality and clinical manifestations of RSV are different from that of influenza infection in adults [123]. Fourth, a clear distinction between annual rates and seasonal rates was not made in several studies, resulting in lower rates in studies that were not limited to the peak respiratory virus season. Lastly, routine clinical practices were highly different by study periods, countries and settings. Testing for RSV was not routinely done in clinical practice especially in out-patient settings, especially before the 2009–10 influenza pandemic or in low- and middle-income countries, leading to the underestimation of RSV rates in most retrospective studies.

Our review had a number of limitations. First, a number of available national surveillance reports related to adult populations, especially from Europe and North America, were not considered in the review as we only included data published in scientific journals. Second, the statistical heterogeneity was expectedly very high (>85% in general). The sample size and estimates varied greatly. In addition, clinical outcomes and study methodology varied greatly including study settings (community clinic or hospital), recruitment (population-based or health care utilisation), case definition (ILI or ARI or SARI or pneumonia with or without chest X-ray confirmation) and diagnostic methods (PCR or latex agglutination), possibly leading to differences in study findings. There were also a number of small studies especially with regard to patients with underlying diseases. These small studies with a sample size of <50 patients may have impacted overall study results.

Finally, the available surveillance and research data on the burden of RSV in adults were much fewer than those in paediatric populations. First, there were very few studies assessing the incidence of RSV in adults in hospitals and in communities; three studies in a hospital setting and three studies in out-patient clinic settings which are much less than those published for paediatric populations. Second, very few studies reported data from Europe and the Americas; most studies were from Asia (24 studies) and Africa (15 studies). RSV surveillance data are usually available in national reports and are mostly limited to children or include all ages without distinction between children and adults. An exploratory analysis of RSV reports through the European Influenza Surveillance System (EISS) and a recent retrospective analysis of RSV reports to European Surveillance System (TESSy) between 2006 and 2010 clearly revealed the need for timely reporting, harmonisation of laboratory techniques and case definitions throughout Europe [123, 124].

This review summarised the overall epidemiologic data related to RSV-associated respiratory infections in adult populations worldwide. The currently available literature suggests that the incidence of RSV is lower in adults than in young children, though elderly patients and those with chronic diseases or transplantation-related immunosuppression are at a higher risk of disease. However, the tremendous heterogeneity in methodology across studies, including case ascertainment and laboratory testing, the inappropriate reliance on influenza surveillance, which does not cover the full spectrum of RSV clinical syndromes, and the inadequacy of existing diagnostic methods to identify RSV cases with low viral loads, all lead to the likely underestimation of disease burden and hamper our ability to draw inferences from between-study comparisons. As new strategies are developed to prevent and treat adult RSV, it will be essential to generate high-quality estimates of disease burden in order to accurately assess the potential public health value of these interventions.

## Supplementary material

For supplementary material accompanying this paper visit https://doi.org/10.1017/S0950268820000400. click here to view supplementary material
