# Supplementary material for: The burden of respiratory syncytial virus in adults: a systematic review and meta-analysis
Source: Epidemiol Infect. 2020 Feb 13;148:e48. doi: 10.1017/S0950268820000400 (PMC7078512; doi:10.1017/S0950268820000400)
Supplement: Supplementary file 1 [file S0950268820000400sup001.docx]

Supplementary tables

Table 1: Characteristics of Studies evaluating incidence of RSV infection in general population

| **Country** | **Study Period** | **Study population** | **Study design** | **Setting** | **Outcomes** | **Diagnostic method** | **Reference** |
| --- | --- | --- | --- | --- | --- | --- | --- |
| Egypt | 2009-2012 | All ages | Population-Based Surveillance | Hospital  Outpatient | ARI ILI | RT-PCR | Rowlinson et al. 2013 [1] |
| Kenya | 2007-2011 | All ages | Population-based surveillance | Outpatient | SARI  ILI | RT-PCR | Bigogo et al. 2013 [2] |
| Kenya | 2009-2012 | All ages | Population-based surveillance | Inpatient Outpatient | SARI ILI | RT-PCR | Emukule et al. 2014 [3] |
| Kenya | 2007-2010 | All ages | Population-based surveillance | Inpatient Outpatient | ARI | RT-PCR | Feikin et al. 2012 [4] |
| Thailand | 2008-2011 | All ages | Population based Surveillance | Hospital | ALRI | RT-PCR | Naorat et al. 2013 [5] |
| USA | 2006-2010 | ≥50 years | Prospective communityhort | Main and Satellite clinics, ED | ARI | Multiplex RT-PCR | McClure et al. 2014 [6] |

ALRI=acute lower respiratory infection; ARI=acute respiratory infection; ED=emergency department; ILI=influenza-like illness; qRT-PCR=quantitative reverse transcription polymerase chain reaction; RT-PCR=reverse transcription polymerase chain reaction; SARI=severe acute respiratory infection

Table 2: Characteristics of studies evaluating the proportion of RSV among respiratory infection in general population

| **Country** | **Period** | **Population** | **Design** | **Setting** | **Outcomes** | **Diagnostic method** | **Reference** |
| --- | --- | --- | --- | --- | --- | --- | --- |
| **Africa** | | | | | | | |
| Egypt | 2009-2012 | All ages | Population-Based Surveillance | Hospital  Outpatient | ARI ILI | RT-PCR | Rowlinson et al. 2013 [1] |
| Kenya | 2007-2011 | All ages | Population-Based Surveillance | Outpatient | SARI  ILI | RT-PCR | Bigogo et al. 2003 [2] |
| Kenya | 2009-2012 | All ages | Population-based surveillance | Inpatient Outpatient | SARI ILI | RT-PCR | Emukule et al. 2014 [3] |
| Kenya | 2007-2010 | All ages | Population-based surveillance | Inpatient Outpatient | ARI | RT-PCR | Feikin et al. 2012 [4] |
| Senegal | 2009-2011 | >50 years | Prospective Descriptive  (Influenza sentinel) | Outpatient | ILI | RT-PCR | Dia et al. 2014 [7] |
| Cameroon | 2009 | All ages | Population based Surveillance  (Influenza sentinel) | Outpatient | ILI | Multiplex RT-PCR | Njouom et al. 2012 [8] |
| Gabon | 2010-2011 | All ages | Population based Surveillance | Health care center, hospital | ILI | Multiplex RT-PCR | Lekana-Douki et al. 2014 [9] |
| South Africa | 1982-1991 | All ages | Active surveillance | Outpatient | ARI | Cell Culture | McAnerney et al. 1994 [10] |
| Latin America | | | | | | | |
| Venezuela | 2006-2010 | All ages | Prospective Surveillance | Outpatient | ILI | RT-PCR | Comach et al. 2012 [11] |
| El Salvador Honduras Nicaragua | 2006-2009 | All ages | Prospective Descriptive | Hospital | ILI | RT-PCR | Laguna-Torres et al. [12] |
| 15 countries | 2008-2009 | ≥65 years | Active surveillance  (Influenza65 trial cohort) | Community / Retirement home | ILI | Multiplex RT-PCR | Falsey et al. 2014 [13] |
| Argentina | 2004 2005 | All ages | Prospective descriptive | Outpatient | ILI | Immunofluorescence | Santamaria et al. 2008 [14] |
| Colombia | 2000-2011 | All ages | Retrospective descriptive | Local health centers, Hospital | ARI | RT-PCR | Barbosa Ramirez et al. 2014 [15] |
| 24 Carribean countries | 2010-2011 | All ages | Surveillance (Caribbean Epidemiology Centre) | Laboratory network | ARI | RT-PCR | Edwards et al. 2013 [16] |
| Guatemala | 2007-2011 | All ages | Surveillance | Hospital | ARI | RT-PCR | Verani et al. 2013 [17] |
| Asia | | | | | | | |
| China | 2010 | All ages | Prospective descriptive | Outpatient | ILI | RT-PCR | Li et al. 2010 [18] |
| China | 2011-2013 | All ages | Prospective descriptive | Hospital | ILI | RT-PCR | Ju et al. 2014 [19] |
| China | 2010-2011 | ≥14 years | Prospective descriptive | ED | ARI | Multiplex RT-PCR | Yu et al. 2012 [20] |
| China | 2005-2007 | ≥14 years | Prospective descriptive | Outpatient | ARI | Multiplex nested RT-PCR | Ren et al. 2009 [21] |
| Nepal | 2011-2014 | Pregnant women | Prospective home-based surveillance | Household | Respiratory illness | RT-PCR | Chu et al. 2016[22] |
| China | 2010-2011 | All ages | Active surveillance | Outpatient | ILI | RT-PCR | Huo et al. 2012 [23] |
| Korea | 2005-2008 | Adults | Retrospective descriptive | Hospital | ARI | Culture | Seo et al. 2014 [24] |
| India | 2011-2012 | All ages | Prospective descriptive | Inpatient Outpatient | ILI, ARI | RT-PCR | Chavan et al. 2015 [25] |
| China | 2005-2010 | ≥15 years | Prospective descriptive | Fever clinic | ARI | Multiplex RT-PCR | Xiang et al. 2013 [26] |
| China | 2009-2013 | All ages | Active surveillance | Sentinel hospitals | ALRI | RT-PCR | Feng et al. 2014 [27] |
| Israel | 1997 | ≥21 years | Prospective descriptive | GP clinic | ARI | EIA | Lieberman et al. 1998 [28] |
| Thailand | 2006-2008 | Adults | Prospective descriptive | Hospital | CAP | Nested RT-PCR | Hara et al. 2011 [29] |
| China | 2009-2010 | ≥14 years | Prospective descriptive | Hospital | ARTI | Multiplex nested RT-PCR | Lu et al. 2013 [30] |
| Thailand | 2008-2011 | All ages | Population based Surveillance | Hospital | ALRI | RT-PCR | Naorat et al. 2013 [5] |
| South Korea | 2010-2012 | Adults | Prospective cohort | Hospital (ICU) | HAP | Multiplex RT-PCR | Hong et al. 2014 [31] |
| Thailand | 2003-2005 | All ages | Active surveillance of pneumonia | Hospital | Pneumonia | RT–PCR | Olsen et al. 2010 [32] |
| India | 2011-2013 | ≥5 years | Prospective descriptive | Hospital | SARI | RT-PCR | Jain et al. 2014 [33] |
| Philippines | 2010-2013 | All ages | Surveillance | Sentinel hospital | ILI | Multiplex RT-PCR | Otomaru et al. 2015 [34] |
| China | 2009-2014 | All ages | Surveillance | Hospital | RTI | RT-PCR | Liao et al. 2015 [35] |
| Taiwan | 2008-2009 | ≥65 years | Active surveillance  (Influenza65 trial cohort) | Community / Retirement home | ILI | Multiplex RT-PCR | Falsey et al. 2014 [13] |
| Kuwait | 3 year study | All ages | Prospective descriptive | Hospital | ARI | PCR | Khadadah et al. 2010 [36] |
| Israel | 2007-2012 | All ages | Descriptive | Hospital | ILI | RT-PCR | Meningher et al. 2014 [37] |
| Lao PDR | 2009-2010 | All ages | Prospective descriptive | Hospital | ALRI | RT-PCR | Sentilhes et al. 2013 [38] |
| Papua New Guinea | 2010 | All ages | Retrospective descriptive | Hospital | ILI | RT-PCR | Kono et al. 2014 [39] |
| China | 2010-2012 | ≥14 years | Prospective descriptive | Hospital | CAP | RT-PCR | Qu et al. 2015 [40] |
| Europe | | | | | | | |
| UK | 2000-2001 | ≥16 years | Active Surveillance | Military Centre | ILI | RT-PCR | O’Shea et al. 2007 [41] |
| Italy | 2004-2005 | All ages | Prospective descriptive | GP clinics | ILI | Multiplex RT-PCR | Rezza et al. 2006 [42] |
| Spain | 2003-2004 | ≥14 years | Prospective descriptive | Hospital | CAP | Multiplex RT-PCR | Angeles et al. 2006 [43] |
| UK | 1999-2000 | All ages | Prospective descriptive | GP clinics | ILI | Multiplex RT-PCR | Wallace et al. 2004 [44] |
| UK | 1995-1998 | All ages | Prospective descriptive | Sentinel GP clinics | ILI | Multiplex RT-PCR | Zambon et al. 2001 [45] |
| UK | 1992­-1994 | ≥60 years | Community survey | Community volunteers | URTI | Complement Fixation | Nicholson et al. 1997 [46] |
| France | 1994-1995 | All ages | Prospective descriptive | GP clinics | ILI | Immunostaining; Cell Culture | Lina et al. 1996 [47] |
| France | 2012-2015 | All ages | Retrospective cohort  (FLUVAC effectiveness study) | Hospital | ILI | RT-PCR | Loubet et al. 2017 [48] |
| Sweden | 1971-1980 | ≥16 years | Retrospective descriptive | Hospital | Pneumonia | Indirect IFA | Vikerfors et al. 1987 [49] |
| Turkey | 2003-2005 | >17 years | Descriptive | Outpatient | CAP | Direct IFA | Koksal et al. 2010 [50] |
| UK | 2009-2010 | All ages | Prospective descriptive | Hospital  GP clinics | ARI | Multiplex RT-PCR | Tanner et al 2012 [51] |
| UK | 1981-1982 | >50 years | Prospective descriptive | Hospital (Geriatric wards) | ARI | Fluorescent antibody technique | Morales et al. 1983 [52] |
| UK | 1974-1980 | >12 years | Descriptive | Hospital | Pneumonia | Complement fixation | White et al. 1981 [53] |
| 8 European countries | 2008-2009 | ≥65 years | Active surveillance  (Influenza65 trial cohort) | Community / Retirement home | ILI | Multiplex RT-PCR | Falsey et al. 2014 [13] |
| US and Canada | | | | | | | |
| USA | 2011-2012 | All ages | Prospective descriptive (EPIC study) | Outpatient | CAP | RT-PCR | Self et al. 2016 [54] |
| USA | 2000-2001 | ≥16 years | Active surveillance ASurveillance | Military Centre | ARI | RT-PCR, culture | O’Shea et al. 2005 [55] |
| USA | 2010-2014 | All ages | Active surveillance | Hospital | SARI | Multiplex PCR | Wansaula et al. 2016 [56] |
| USA | 2009-2010 | ≥18 years | Retrospective descriptive | Hospital | RVI | xTAG respiratory viral panel | Walker et al. 2014 [57] |
| USA | 1990-1992 | ≥18 years | Prospective cohort | Hospital | CAP | Indirect EIA | Dowell et al. 1996 [58] |
| USA | 2009-2010 | ≥18 years | Retrospective descriptive (Influenza effectiveness study) | Hospital  ED | ARI | RT-PCR | Widmer et al. 2014 [59] |
| USA | 1989-1992 | ≥65 years | (Influenza surveillance) | Hospital | ILI  Acute cardiopulmonary condition | Culture EIA | Falsey et al. 1995 [60] |
| USA | 2002 | ≥18 years | Prospective descriptive | ED | ARI | RT-PCR | Louie et al. 2005 [61] |
| USA | 2012 | All ages | Prospective descriptive (Influenza effectiveness study) | Outpatient | MA-ARI | Multiplex RT-PCR | Zimmerman et al. 2014 [62] |
| USA | 1975-1995 | 18-60 years | Surveillance | Hospital | RVI | Culture | Hall et al. 2001 [63] |
| USA | 1999-2003 | ≥65 years | Prospective cohorts | Community Hospital | ARI | RT-PCR | Falsey et al. 2005 [64] |
| USA | 2004-2010 | ≥50 years | Prospective descriptive | Inpatient  Outpatient | MA-ARI | Multiplex RT-PCR | Sundaram et al. 2014 [65] |
| USA | 2006-2010 | ≥50 years | Prospective community cohort | Main clinic  Satellite clinic  Emergency department | MA-ARI | Multiplex RT-PCR | McClure et al. 2014 [6] |
| USA | 2006-2009 | ≥50 years | Prospective  (Influenza VE study) | Hospital | ARI | RT-PCR | Widmer et al. 2012 [66] |
| Canada | 2004-2006 | Adults | Prospective descriptive | Hospital | CAP | DFA Testing | Johnstone et al. 2008 [67] |

Table 3: Stratified analysis for the proportion of RSV associated ARI in Asia

| Variable | Summary estimate | Lower 95% CI | Upper 95% CI | I^2^ | Number of estimates | Inter-group heterogeneity (p-value<0.05) |  |
| --- | --- | --- | --- | --- | --- | --- | --- |
| *Age Group* |  |  |  |  |  | 0.0000 | |
| <50 years | 0.01 | 0.00 | 0.02 | 92.69 | 10 |  | |
| >=50 years | 0.02 | 0.01 | 0.03 | 90.93 | 7 |  | |
| All Ages | 0.10 | 0.07 | 0.15 | 99.16 | 19 |  | |
| All Adults | 0.01 | 0 | 0.02 | 82.36 | 16 |  | |
| ***Study Period*** |  |  |  |  |  | 0.4337 | |
| Before 2000 | - | - | - | - | - |  | |
| 2000-2010 | 0.04 | 0.02 | 0.06 | 98.69 | 30 |  | |
| Post 2010 | 0.04 | 0.02 | 0.07 | 99.09 | 21 |  | |
| ***Illness Definition*** |  |  |  |  |  | 0.0000 | |
| SARI | * | * | * | * |  |  | |
| CAP | 0.03 | 0.02 | 0.04 | 23.14 | 6 |  | |
| ILI | 0.04 | 0.02 | 0.07 | 94.99 | 13 |  | |
| ARI | 0.02 | 0 | 0.03 | 99.10 | 20 |  | |
| Miscellaneous | 0.09 | 0.03 | 0.16 | 99.48 | 11 |  | |
| ***Diagnostic Method*** |  |  |  |  |  | 0.0150 | |
| Multiplex PCR | 0.04 | 0.02 | 0.07 | 97.98 | 15 |  | |
| Other PCR | 0.05 | 0.03 | 0.07 | 99.35 | 28 |  | |
| DFA/IIF/ELIZA/EIA | - | - | - | - | - |  | |
| Culture | * | * | * | * | * |  | |
| Other | * | * | * | * | * |  | |
| Mixed | * | * | * | * | * |  | |

Note: '-' not calculated by program, low number of estimates in the group, no variation considered between the variables as all estimates from the same study; ‘*’ estimates not provided all the estimates in the group from same study

Table 4: Meta-regression results for Asia

| Variable | **Coefficient** | **Standard error** | **p-value** |
| --- | --- | --- | --- |
| *Age Group* | 0.010 | 0.010 | 0.302 |
| *Study Period* | 0.018 | 0.021 | 0.405 |
| *Illness Definition* | 0.014 | 0.011 | 0.210 |
| *Diagnostic Method* | -0.005 | 0.009 | 0.550 |

Table 5: Meta-regression results for the United States and Canada

| Variable | Coefficient | Standard error | p-value |
| --- | --- | --- | --- |
| Age Group | -0.023 | 0.010 | 0.030 |
| Study Period | -0.016 | 0.022 | 0.480 |
| Illness Definition | 0.013 | 0.009 | 0.152 |
| Diagnostic Method | -0.005 | 0.063 | 0.094 |

Table 6: Study characteristics for proportion of RSV in population with underlying conditions or diseases

| **Country** | **Study Period** | **Population** | **Design** | **Setting** | **Outcomes** | **Diagnostic method** | **Reference** |
| --- | --- | --- | --- | --- | --- | --- | --- |
| Australia | 1993-1994 | Adults ; Asthma | Case control | Hospital | AE-asthma | Serology / culture | Teichtahl et al. 1997 [68] |
| Australia | 2003-2005 | >50 years; COPD | Prospective cohort | Hospital | AE-COPD | Multiplex PCR | Hutchinson te al. 2007 [69] |
| Australia | 2009-2012 | Multiple myeloma | Retrospective cohort | Cancer center | VRI | multiplex PCR | Teh et al. 2015 [70] |
| Canada | 2007-2008 | COPD | Prospective cohort | Hospital  ED | AE-COPD | RT-PCR | Kherad et al. 2010 [71] |
| Canada (Quebec) | 2002-2003 | >50 years of age, COPD | Prospective cohort | Hospital | AE-COPD | RT-PCR | De Serres et al. 2009 [72] |
| Europe | 1997-1998 | HSCT | Prospective cohort | BMT centers | ARI | Not described | Ljungman et al. 2001 [73] |
| France | 2002-2004 | Chronic cardiac or pulmonary disorder | Descriptive | CCU | Acute respiratory or cardiac failure | RT-PCR | Carrat et al. 2006 [74] |
| Greece | 2008-2009 | ≥18 years ; confirmed COPD | Prospective descriptive | Hospital | AE-COPD | RT-PCR | Dimopoulos et al. 2012[75] |
| Hong Kong | 2004-2005 | COPD | Prospective cohort | Hospital | AE-COPD | Culture, multiplex PCR | Ko et al. 2007[76] |
| Iran | 2010-2012 | Adults, COPD | Case-control | Hospital | AE-COPD | PCR | Hosseini et al. 2015[77] |
| Kenya | 2007-2010 | HIV + | Surveillance | Inpatient  Outpatient | ARI | RT-PCR | Feikin et al. 2012 [4] |
| Spain | 1999-2003 | Adult, HSCT | Prospective cohort | Hospital | ARI | RT-PCR | Martino et al. 2005 [78] |
| Sweden | 2000-2007 | Allogenic HSCT | Retrospective chart review | Hospital | ARI | Not described | Avetisyan et al. 2009 [79] |
| Sweden | NA | Cardiovascular disease, Chronic lung disease,  Chronic alcoholism, Diabetes mellitus, Malignancy, Splenectomy, Rheumatoid arthritis, Drug abuse, Ankylosing spondylitis, SLE | Descriptive | Hospital | CAP | Indirect IFA | Berntsson et al. 1985 [80] |
| UK | 1981-1982 | Elderly, geriatric long stay patients | Prospective descriptive | Geriatric wards | ARI | Culture | Morales et al. 1983 [52] |
| UK | 1990-1992 | Adults, Asthma | Prospective descriptive | Hospital | AE-asthma | Serology / culture | Nicholson et al. 1993 [81] |
| UK | NA | COPD | Prosepctive cohort | Outpatient clincic | AE-COPD | PCR | Seemungal et al. 2001 [82] |
| USA | 1989-1990 | ≥65 years, institutionnalized patients | Prospective descriptive | Home for elderly | ARI | Culture | Falsey et al. 1992 [83] |
| USA | 1992-1993 | Elderly, senior daycare center | Prospective descriptive | Senior day care center | ARI | Culture | Falsey et al. 1995 [84] |
| USA | 1992-1994 | Adults, BMT | Prospective descriptive | Cancer center | ARI | Culture | Whimbey et al. 1996 [85] |
| USA | 1993-1994 | Leucemia | Prospective descriptive | Cancer center | ARI | Culture  Indirect IFA | Whimbey et al. 1995 [86] |
| USA | 1992-1997 | Adult, lung transplant | Retrospective cohort | Cancer center | RVI | Not described | Palmer et al. 1998 [87] |
| USA | 1994-1999 | Adult, allogenic HSCT | Retrospective descriptive | Cancer center | RSV infection | DFA / Pack EIA | Small et al. 2002 [88] |
| USA | 1996-1998 | COPD or CHF | Prospective cohort | dwelleing community | ARI | Culture  EIA | Walsh et al. 1999 [89] |
| USA | 1997-1998 | Cancer patients receiving cytotoxic chemotherapy with or without BMT | Prospective cohort | Hospital | RSV infection | IFA / culture | Anaissie et al. 2004 [90] |
| USA | 1993-2006 | Pediatric HSCT, SOT and chemotherapy | Retrospective cohort | Hospital | RVI | DFA / PCR / Culture | Lo t al. 2013 [91] |
| USA | 1999-2003 | ≥65 years, cardiopulmonary disease | Prospective cohort | Hospital | ARI | RT-PCR | Falsey et al. 2005 [92] |
| USA | 2000-2004 | Adults, HSCT | Prospective descriptive | Hospital | RVI | RT-PCR | Peck et al. 2007 [93] |
| USA | 2004-2010 | Adults; COPD/ liver/renal diseasaes, CHF | Prospective | Inpatient  Outpatient | ARI | PCR | Sundaram et al. 2014 [65] |
| USA | 2002 | Multiple diseases (Allergy, COPD, Heart disease, Liver disease, Diabetes, Cancer, Asthma) | Prospective, descriptive | ED | ARI | PCR  Culture | Louie et al. 2005 [61] |
| USA | 2002-2003 | COPD | Prospective descriptive | Hospital | AE-COPD | RT-PCR | Martinello et al. 5006 [94] |
| USA | 2003-2004 | COPD | Prospective  cohort | ED | AE-COPD | PCR | Camargo et al. 2008 [95] |
| USA | 2009-2010 | Multiple diseases (Lung disease, Liver disease, Renal disease, Diabetes, Cardiovascular disease, Immunocompromised (any cause)) | Retrospective chart review | Hospital | RVI | RVP ProFlu+ | Walker et al. 2014 [57] |
| USA | 2009-2010 | Adults, Lives alone, Lives with family In nursing facility, Chronic illnesses, Cardiovascular disease, Pulmonary disease, Diabetes, Immunodeficiency , Exposure to tobacco smoke | Retrospective descriptive (Influenza effectiveness study) | Hospital  ED | ARI | RT-PCR | Widmer et al. 2014 [59] |
| USA | 2010-2014 | Hypertension, Metabolic disorder, Chronic lung disease, Cardiac disease, Current smoker, Immunosuppression, Morbid obesity  Neuromuscular disease | Active surveillance | Hospital | SARI | Multiplex PCR | Wansaula et al. 2016 [56] |

1. Rowlinson E, Dueger E, Taylor T, Mansour A, Van Beneden C, Abukela M, et al. Incidence and clinical features of respiratory syncytial virus infections in a population-based surveillance site in the Nile Delta Region. The Journal of Infectious Diseases. 2013;208 Suppl 3:S189-96. doi: 10.1093/infdis/jit457. PubMed PMID: 24265478.

2. Bigogo GM, Breiman RF, Feikin DR, Audi AO, Aura B, Cosmas L, et al. Epidemiology of respiratory syncytial virus infection in rural and urban Kenya. The Journal of Infectious Diseases. 2013;208 Suppl 3:S207-16. Epub 2013/12/07. doi: 10.1093/infdis/jit489. PubMed PMID: 24265480.

3. Emukule GO, Khagayi S, McMorrow ML, Ochola R, Otieno N, Widdowson MA, et al. The burden of influenza and RSV among inpatients and outpatients in rural western Kenya, 2009-2012. PloS one. 2014;9(8):e105543. Epub 2014/08/19. doi: 10.1371/journal.pone.0105543. PubMed PMID: 25133576; PubMed Central PMCID: PMCPMC4136876.

4. Feikin DR, Njenga MK, Bigogo G, Aura B, Aol G, Audi A, et al. Etiology and Incidence of viral and bacterial acute respiratory illness among older children and adults in rural western Kenya, 2007-2010. PloS one. 2012;7(8):e43656. Epub 2012/09/01. doi: 10.1371/journal.pone.0043656. PubMed PMID: 22937071; PubMed Central PMCID: PMCPMC3427162.

5. Naorat S, Chittaganpitch M, Thamthitiwat S, Henchaichon S, Sawatwong P, Srisaengchai P, et al. Hospitalizations for acute lower respiratory tract infection due to respiratory syncytial virus in Thailand, 2008-2011. The Journal of Infectious Diseases. 2013;208 Suppl 3:S238-45. Epub 2013/12/07. doi: 10.1093/infdis/jit456. PubMed PMID: 24265483.

6. McClure DL, Kieke BA, Sundaram ME, Simpson MD, Meece JK, Sifakis F, et al. Seasonal incidence of medically attended respiratory syncytial virus infection in a community cohort of adults >/=50 years old. PloS one. 2014;9(7):e102586. doi: 10.1371/journal.pone.0102586. PubMed PMID: 25025344; PubMed Central PMCID: PMC4099308.

7. Dia N, Richard V, Kiori D, Cisse el HA, Sarr FD, Faye A, et al. Respiratory viruses associated with patients older than 50 years presenting with ILI in Senegal, 2009 to 2011. BMC Infectious Diseases. 2014;14:189. Epub 2014/04/10. doi: 10.1186/1471-2334-14-189. PubMed PMID: 24712515; PubMed Central PMCID: PMCPMC4020602.

8. Njouom R, Yekwa EL, Cappy P, Vabret A, Boisier P, Rousset D. Viral etiology of influenza-like illnesses in Cameroon, January-December 2009. The Journal of Infectious Diseases. 2012;206 Suppl 1:S29-35. Epub 2012/11/28. doi: 10.1093/infdis/jis573. PubMed PMID: 23169968.

9. Lekana-Douki SE, Nkoghe D, Drosten C, Ngoungou EB, Drexler JF, Leroy EM. Viral etiology and seasonality of influenza-like illness in Gabon, March 2010 to June 2011. BMC Infectious Diseases. 2014;14:373. Epub 2014/07/09. doi: 10.1186/1471-2334-14-373. PubMed PMID: 25000832; PubMed Central PMCID: PMCPMC4107952.

10. McAnerney JM, Johnson S, Schoub BD. Surveillance of respiratory viruses. A 10-year laboratory-based study. South African Medical Journal = Suid-Afrikaanse tydskrif vir geneeskunde. 1994;84(8 Pt 1):473-7. Epub 1994/08/01. PubMed PMID: 7825079.

11. Comach G, Teneza-Mora N, Kochel TJ, Espino C, Sierra G, Camacho DE, et al. Sentinel surveillance of influenza-like illness in two hospitals in Maracay, Venezuela: 2006-2010. PloS one. 2012;7(9):e44511. Epub 2012/09/18. doi: 10.1371/journal.pone.0044511. PubMed PMID: 22984519; PubMed Central PMCID: PMCPMC3439372.

12. Laguna-Torres VA, Sanchez-Largaespada JF, Lorenzana I, Forshey B, Aguilar P, Jimenez M, et al. Influenza and other respiratory viruses in three Central American countries. Influenza and Other Respiratory Viruses. 2011;5(2):123-34. Epub 2011/02/11. doi: 10.1111/j.1750-2659.2010.00182.x. PubMed PMID: 21306576; PubMed Central PMCID: PMCPMC4942008.

13. Falsey AR, McElhaney JE, Beran J, van Essen GA, Duval X, Esen M, et al. Respiratory syncytial virus and other respiratory viral infections in older adults with moderate to severe influenza-like illness. The Journal of Infectious Diseases. 2014;209(12):1873-81. Epub 2014/02/01. doi: 10.1093/infdis/jit839. PubMed PMID: 24482398; PubMed Central PMCID: PMCPMC4038137.

14. Santamaria C, Uruena A, Videla C, Suarez A, Ganduglia C, Carballal G, et al. Epidemiological study of influenza virus infections in young adult outpatients from Buenos Aires, Argentina. Influenza and Other Respiratory Viruses. 2008;2(4):131-4. Epub 2009/05/21. doi: 10.1111/j.1750-2659.2008.00048.x. PubMed PMID: 19453464; PubMed Central PMCID: PMCPMC4634226.

15. Barbosa Ramirez J, Pulido Dominguez P, Rey Benito G, Mendez Rico J, Castellanos J, Paez Martinez A. Human respiratory syncytial virus and metapneumovirus in patients with acute respiratory infection in Colombia, 2000 - 2011. Revista Panamericana de Salud Publica = Pan American Journal of Public Health. 2014;36(2):101-9. Epub 2014/10/28. PubMed PMID: 25345531.

16. Edwards L, Boisson E, Nathaniel-Girdharrie S, Morris-Glasgow V. Distribution of influenza and other acute respiratory viruses during the first year after the 2009-2010 influenza pandemic in the English- and Dutch-speaking Caribbean countries. Influenza and Other Respiratory Viruses. 2013;7(6):1062-9. Epub 2013/06/12. doi: 10.1111/irv.12126. PubMed PMID: 23745666; PubMed Central PMCID: PMCPMC4634279.

17. Verani JR, McCracken J, Arvelo W, Estevez A, Lopez MR, Reyes L, et al. Surveillance for hospitalized acute respiratory infection in Guatemala. PloS one. 2013;8(12):e83600. Epub 2014/01/07. doi: 10.1371/journal.pone.0083600. PubMed PMID: 24391792; PubMed Central PMCID: PMCPMC3877070.

18. Li H, Wei Q, Tan A, Wang L. Epidemiological analysis of respiratory viral etiology for influenza-like illness during 2010 in Zhuhai, China. Virology journal. 2013;10:143. Epub 2013/05/09. doi: 10.1186/1743-422x-10-143. PubMed PMID: 23651577; PubMed Central PMCID: PMCPMC3655035.

19. Ju X, Fang Q, Zhang J, Xu A, Liang L, Ke C. Viral etiology of influenza-like illnesses in Huizhou, China, from 2011 to 2013. Archives of virology. 2014;159(8):2003-10. Epub 2014/03/13. doi: 10.1007/s00705-014-2035-1. PubMed PMID: 24610554.

20. Yu X, Lu R, Wang Z, Zhu N, Wang W, Julian D, et al. Etiology and clinical characterization of respiratory virus infections in adult patients attending an emergency department in Beijing. PloS one. 2012;7(2):e32174. Epub 2012/03/06. doi: 10.1371/journal.pone.0032174. PubMed PMID: 22389685; PubMed Central PMCID: PMCPMC3289638.

21. Ren L, Gonzalez R, Wang Z, Xiang Z, Wang Y, Zhou H, et al. Prevalence of human respiratory viruses in adults with acute respiratory tract infections in Beijing, 2005-2007. Clinical Microbiology and Infection : the official publication of the European Society of Clinical Microbiology and Infectious Diseases. 2009;15(12):1146-53. Epub 2009/05/22. doi: 10.1111/j.1469-0691.2009.02746.x. PubMed PMID: 19456830.

22. Chu HY, Katz J, Tielsch J, Khatry SK, Shrestha L, LeClerq SC, et al. Clinical Presentation and Birth Outcomes Associated with Respiratory Syncytial Virus Infection in Pregnancy. PloS one. 2016;11(3):e0152015. Epub 2016/04/01. doi: 10.1371/journal.pone.0152015. PubMed PMID: 27031702; PubMed Central PMCID: PMCPMC4816499.

23. Huo X, Qin Y, Qi X, Zu R, Tang F, Li L, et al. Surveillance of 16 respiratory viruses in patients with influenza-like illness in Nanjing, China. Journal of Medical Virology. 2012;84(12):1980-4. Epub 2012/10/20. doi: 10.1002/jmv.23401. PubMed PMID: 23080506.

24. Seo YB, Cheong HJ, Song JY, Noh JY, Kim IS, Song DJ, et al. Epidemiologic differences of four major respiratory viruses between children, adolescents, and adults in Korea. Journal of infection and chemotherapy : Official Journal of the Japan Society of Chemotherapy. 2014;20(11):672-7. Epub 2014/09/03. doi: 10.1016/j.jiac.2013.07.009. PubMed PMID: 25179390.

25. Chavan RD, Kothari ST, Zunjarrao K, Chowdhary AS. Surveillance of acute respiratory infections in Mumbai during 2011-12. Indian Journal of Medical Microbiology. 2015;33(1):43-50. Epub 2015/01/07. doi: 10.4103/0255-0857.148376. PubMed PMID: 25560001.

26. Xiang Z, Gonzalez R, Ren L, Xiao Y, Chen L, Zhang J, et al. Prevalence and clinical characteristics of human respiratory syncytial virus in Chinese adults with acute respiratory tract infection. Journal of Medical Virology. 2013;85(2):348-53. Epub 2012/11/30. doi: 10.1002/jmv.23467. PubMed PMID: 23192884.

27. Feng L, Li Z, Zhao S, Nair H, Lai S, Xu W, et al. Viral etiologies of hospitalized acute lower respiratory infection patients in China, 2009-2013. PloS one. 2014;9(6):e99419. Epub 2014/06/20. doi: 10.1371/journal.pone.0099419. PubMed PMID: 24945280; PubMed Central PMCID: PMCPMC4063718.

28. Lieberman D, Shvartzman P, Lieberman D, Ben-Yaakov M, Lazarovich Z, Hoffman S, et al. Etiology of respiratory tract infection in adults in a general practice setting. European Journal of Clinical Microbiology & Infectious Diseases : official publication of the European Society of Clinical Microbiology. 1998;17(10):685-9. Epub 1998/12/29. PubMed PMID: 9865980.

29. Hara K, Yahara K, Gotoh K, Nakazono Y, Kashiwagi T, Imamura Y, et al. Clinical study concerning the relationship between community-acquired pneumonia and viral infection in northern Thailand. Internal Medicine (Tokyo, Japan). 2011;50(9):991-8. Epub 2011/05/03. PubMed PMID: 21532221.

30. Lu Y, Tong J, Pei F, Yang Y, Xu D, Ji M, et al. Viral aetiology in adults with acute upper respiratory tract infection in Jinan, Northern China. Clinical & Developmental Immunology. 2013;2013:869521. Epub 2013/05/22. doi: 10.1155/2013/869521. PubMed PMID: 23690828; PubMed Central PMCID: PMCPMC3649347.

31. Hong HL, Hong SB, Ko GB, Huh JW, Sung H, Do KH, et al. Viral infection is not uncommon in adult patients with severe hospital-acquired pneumonia. PloS one. 2014;9(4):e95865. Epub 2014/04/23. doi: 10.1371/journal.pone.0095865. PubMed PMID: 24752070; PubMed Central PMCID: PMCPMC3994115.

32. Olsen SJ, Thamthitiwat S, Chantra S, Chittaganpitch M, Fry AM, Simmerman JM, et al. Incidence of respiratory pathogens in persons hospitalized with pneumonia in two provinces in Thailand. Epidemiology and Infection. 2010;138(12):1811-22. Epub 2010/04/01. doi: 10.1017/s0950268810000646. PubMed PMID: 20353622.

33. Jain B, Singh AK, Dangi T, Agarwal A, Verma AK, Dwivedi M, et al. High prevalence of human metapneumovirus subtype B in cases presenting as severe acute respiratory illness: an experience at tertiary care hospital. The Clinical Respiratory Journal. 2014;8(2):225-33. Epub 2013/10/18. doi: 10.1111/crj.12064. PubMed PMID: 24131502.

34. Otomaru H, Kamigaki T, Tamaki R, Opinion J, Santo A, Daya E, et al. Influenza and other respiratory viruses detected by influenza-like illness surveillance in Leyte Island, the Philippines, 2010-2013. PloS one. 2015;10(4):e0123755. Epub 2015/04/22. doi: 10.1371/journal.pone.0123755. PubMed PMID: 25893441; PubMed Central PMCID: PMCPMC4404362.

35. Liao X, Hu Z, Liu W, Lu Y, Chen D, Chen M, et al. New Epidemiological and Clinical Signatures of 18 Pathogens from Respiratory Tract Infections Based on a 5-Year Study. PloS one. 2015;10(9):e0138684. Epub 2015/09/26. doi: 10.1371/journal.pone.0138684. PubMed PMID: 26406339; PubMed Central PMCID: PMCPMC4583381.

36. Khadadah M, Essa S, Higazi Z, Behbehani N, Al-Nakib W. Respiratory syncytial virus and human rhinoviruses are the major causes of severe lower respiratory tract infections in Kuwait. Journal of Medical Virology. 2010;82(8):1462-7. Epub 2010/06/24. doi: 10.1002/jmv.21823. PubMed PMID: 20572084.

37. Meningher T, Hindiyeh M, Regev L, Sherbany H, Mendelson E, Mandelboim M. Relationships between A(H1N1)pdm09 influenza infection and infections with other respiratory viruses. Influenza and Other Respiratory Viruses. 2014;8(4):422-30. Epub 2014/04/05. doi: 10.1111/irv.12249. PubMed PMID: 24698156; PubMed Central PMCID: PMCPMC4181801.

38. Sentilhes AC, Choumlivong K, Celhay O, Sisouk T, Phonekeo D, Vongphrachanh P, et al. Respiratory virus infections in hospitalized children and adults in Lao PDR. Influenza and Other Respiratory Viruses. 2013;7(6):1070-8. Epub 2013/06/26. doi: 10.1111/irv.12135. PubMed PMID: 23796419; PubMed Central PMCID: PMCPMC4634274.

39. Kono J, Jonduo MH, Omena M, Siba PM, Horwood PF. Viruses associated with influenza-like-illnesses in Papua New Guinea, 2010. Journal of Medical Virology. 2014;86(5):899-904. Epub 2013/10/19. doi: 10.1002/jmv.23786. PubMed PMID: 24136362.

40. Qu JX, Gu L, Pu ZH, Yu XM, Liu YM, Li R, et al. Viral etiology of community-acquired pneumonia among adolescents and adults with mild or moderate severity and its relation to age and severity. BMC Infectious Diseases. 2015;15:89. Epub 2015/03/27. doi: 10.1186/s12879-015-0808-0. PubMed PMID: 25812108; PubMed Central PMCID: PMCPMC4342096.

41. O'Shea MK, Pipkin C, Cane PA, Gray GC. Respiratory syncytial virus: an important cause of acute respiratory illness among young adults undergoing military training. Influenza and Other Respiratory Viruses. 2007;1(5-6):193-7. Epub 2008/10/11. doi: 10.1111/j.1750-2659.2007.00029.x. PubMed PMID: 18846262; PubMed Central PMCID: PMCPMC2564797.

42. Rezza G, Valdarchi C, Puzelli S, Ciotti M, Farchi F, Fabiani C, et al. Respiratory viruses and influenza-like illness: a survey in the area of Rome, winter 2004-2005. Eurosurveillance. 2006;11(10):251-3. Epub 2006/11/30. PubMed PMID: 17130656.

43. Angeles Marcos M, Camps M, Pumarola T, Antonio Martinez J, Martinez E, Mensa J, et al. The role of viruses in the aetiology of community-acquired pneumonia in adults. Antiviral Therapy. 2006;11(3):351-9. Epub 2006/06/09. PubMed PMID: 16759052.

44. Wallace LA, Collins TC, Douglas JD, McIntyre S, Millar J, Carman WF. Virological surveillance of influenza-like illness in the community using PCR and serology. Journal of Clinical Virology : the official publication of the Pan American Society for Clinical Virology. 2004;31(1):40-5. Epub 2004/08/04. doi: 10.1016/j.jcv.2003.12.003. PubMed PMID: 15288612.

45. Zambon MC, Stockton JD, Clewley JP, Fleming DM. Contribution of influenza and respiratory syncytial virus to community cases of influenza-like illness: an observational study. Lancet. 2001;358(9291):1410-6. Epub 2001/11/14. PubMed PMID: 11705487.

46. Nicholson KG, Kent J, Hammersley V, Cancio E. Acute viral infections of upper respiratory tract in elderly people living in the community: comparative, prospective, population based study of disease burden. British Medical Journal. 1997;315(7115):1060-4. Epub 1997/11/21. PubMed PMID: 9366736; PubMed Central PMCID: PMCPMC2127683.

47. Lina B, Valette M, Foray S, Luciani J, Stagnara J, See DM, et al. Surveillance of community-acquired viral infections due to respiratory viruses in Rhone-Alpes (France) during winter 1994 to 1995. Journal of Clinical Microbiology. 1996;34(12):3007-11. Epub 1996/12/01. PubMed PMID: 8940439; PubMed Central PMCID: PMCPMC229450.

48. Loubet P, Lenzi N, Valette M, Foulongne V, Krivine A, Houhou N, et al. Clinical characteristics and outcome of respiratory syncytial virus infection among adults hospitalized with influenza-like illness in France. Clinical Microbiology and Infection : the official publication of the European Society of Clinical Microbiology and Infectious Diseases. 2017;23(4):253-9. doi: 10.1016/j.cmi.2016.11.014. PubMed PMID: 27903461.

49. Vikerfors T, Grandien M, Olcen P. Respiratory syncytial virus infections in adults. The American Review of Respiratory Disease. 1987;136(3):561-4. Epub 1987/09/01. doi: 10.1164/ajrccm/136.3.561. PubMed PMID: 3631728.

50. Koksal I, Ozlu T, Bayraktar O, Yilmaz G, Bulbul Y, Oztuna F, et al. Etiological agents of community-acquired pneumonia in adult patients in Turkey; a multicentric, cross-sectional study. Tuberkuloz ve toraks. 2010;58(2):119-27. Epub 2010/09/25. PubMed PMID: 20865563.

51. Tanner H, Boxall E, Osman H. Respiratory viral infections during the 2009-2010 winter season in Central England, UK: incidence and patterns of multiple virus co-infections. European Journal of Clinical Microbiology & Infectious Diseases : official publication of the European Society of Clinical Microbiology. 2012;31(11):3001-6. doi: 10.1007/s10096-012-1653-3. PubMed PMID: 22678349.

52. Morales F, Calder MA, Inglis JM, Murdoch PS, Williamson J. A study of respiratory infections in the elderly to assess the role of respiratory syncytial virus. The Journal of Infection. 1983;7(3):236-47. Epub 1983/11/01. PubMed PMID: 6663083.

53. White RJ, Blainey AD, Harrison KJ, Clarke SK. Causes of pneumonia presenting to a district general hospital. Thorax. 1981;36(8):566-70. Epub 1981/08/01. PubMed PMID: 7314031; PubMed Central PMCID: PMCPMC471626.

54. Self WH, Williams DJ, Zhu Y, Ampofo K, Pavia AT, Chappell JD, et al. Respiratory Viral Detection in Children and Adults: Comparing Asymptomatic Controls and Patients With Community-Acquired Pneumonia. The Journal of Infectious Diseases. 2016;213(4):584-91. Epub 2015/07/17. doi: 10.1093/infdis/jiv323. PubMed PMID: 26180044; PubMed Central PMCID: PMCPMC4721902.

55. O'Shea MK, Ryan MA, Hawksworth AW, Alsip BJ, Gray GC. Symptomatic respiratory syncytial virus infection in previously healthy young adults living in a crowded military environment. Clinical Infectious Diseases : an official publication of the Infectious Diseases Society of America. 2005;41(3):311-7. Epub 2005/07/12. doi: 10.1086/431591. PubMed PMID: 16007526.

56. Wansaula Z, Olsen SJ, Casal MG, Golenko C, Erhart LM, Kammerer P, et al. Surveillance for severe acute respiratory infections in Southern Arizona, 2010-2014. Influenza and Other Respiratory Viruses. 2016;10(3):161-9. Epub 2015/11/22. doi: 10.1111/irv.12360. PubMed PMID: 26590069; PubMed Central PMCID: PMCPMC4814863.

57. Walker E, Ison MG. Respiratory viral infections among hospitalized adults: experience of a single tertiary healthcare hospital. Influenza and Other Respiratory Viruses. 2014;8(3):282-92. Epub 2014/02/05. doi: 10.1111/irv.12237. PubMed PMID: 24490751; PubMed Central PMCID: PMCPMC4181476.

58. Dowell SF, Anderson LJ, Gary HE, Erdman DD, Plouffe JF, File TM, et al. Respiratory syncytial virus is an important cause of community-acquired lower respiratory infection among hospitalized adults. Journal of Infectious Diseases. 1996;174(3):456-62.

59. Widmer K, Griffin MR, Zhu Y, Williams JV, Talbot HK. Respiratory syncytial virus- and human metapneumovirus-associated emergency department and hospital burden in adults. Influenza and Other Respiratory Viruses. 2014;8(3):347-52. doi: 10.1111/irv.12234. PubMed PMID: 24512531; PubMed Central PMCID: PMC3984605.

60. Falsey AR, Cunningham CK, Barker WH, Kouides RW, Yuen JB, Menegus M, et al. Respiratory syncytial virus and influenza A infections in the hospitalized elderly. Journal of Infectious Diseases. 1995;172(2):389-94.

61. Louie JK, Hacker JK, Gonzales R, Mark J, Maselli JH, Yagi S, et al. Characterization of viral agents causing acute respiratory infection in a San Francisco University Medical Center Clinic during the influenza season. Clinical infectious diseases : an official publication of the Infectious Diseases Society of America. 2005;41(6):822-8. Epub 2005/08/19. doi: 10.1086/432800. PubMed PMID: 16107980.

62. Zimmerman RK, Rinaldo CR, Nowalk MP, Gk B, Thompson MG, Moehling KK, et al. Influenza and other respiratory virus infections in outpatients with medically attended acute respiratory infection during the 2011-12 influenza season. Influenza and Other Respiratory Viruses. 2014;8(4):397-405. Epub 2014/05/24. doi: 10.1111/irv.12247. PubMed PMID: 24852890; PubMed Central PMCID: PMCPMC4057994.

63. Hall CB, Long CE, Schnabel KC. Respiratory syncytial virus infections in previously healthy working adults. Clinical Infectious Diseases. 2001;33(6):792-6.

64. Falsey AR, Hennessey PA, Formica MA, Cox C, Walsh EE. Respiratory syncytial virus infection in elderly and high-risk adults. New England Journal of Medicine. 2005;352(17):1749-59.

65. Sundaram ME, Meece JK, Sifakis F, Gasser RA, Jr., Belongia EA. Medically attended respiratory syncytial virus infections in adults aged >/= 50 years: clinical characteristics and outcomes. Clinical infectious diseases : an official publication of the Infectious Diseases Society of America. 2014;58(3):342-9. Epub 2013/11/23. doi: 10.1093/cid/cit767. PubMed PMID: 24265361.

66. Widmer K, Zhu Y, Williams JV, Griffin MR, Edwards KM, Talbot HK. Rates of hospitalizations for respiratory syncytial virus, human metapneumovirus, and influenza virus in older adults. The Journal of Infectious Diseases. 2012;206(1):56-62. Epub 2012/04/25. doi: 10.1093/infdis/jis309. PubMed PMID: 22529314; PubMed Central PMCID: PMCPMC3415933.

67. Johnstone J, Majumdar SR, Fox JD, Marrie TJ. Viral infection in adults hospitalized with community-acquired pneumonia: prevalence, pathogens, and presentation. Chest. 2008;134(6):1141-8. Epub 2008/08/12. doi: 10.1378/chest.08-0888. PubMed PMID: 18689592.

68. Teichtahl H, Buckmaster N, Pertnikovs E. The incidence of respiratory tract infection in adults requiring hospitalization for asthma. Chest. 1997;112(3):591-6. Epub 1997/10/07. PubMed PMID: 9315789.

69. Hutchinson AF, Ghimire AK, Thompson MA, Black JF, Brand CA, Lowe AJ, et al. A community-based, time-matched, case-control study of respiratory viruses and exacerbations of COPD. Respiratory Medicine. 2007;101(12):2472-81. Epub 2007/09/08. doi: 10.1016/j.rmed.2007.07.015. PubMed PMID: 17822891.

70. Teh BW, Worth LJ, Harrison SJ, Thursky KA, Slavin MA. Risks and burden of viral respiratory tract infections in patients with multiple myeloma in the era of immunomodulatory drugs and bortezomib: experience at an Australian Cancer Hospital. Supportive care in cancer : official journal of the Multinational Association of Supportive Care in Cancer. 2015;23(7):1901-6. Epub 2014/12/10. doi: 10.1007/s00520-014-2550-3. PubMed PMID: 25487843.

71. Kherad O, Kaiser L, Bridevaux PO, Sarasin F, Thomas Y, Janssens JP, et al. Upper-respiratory viral infection, biomarkers, and COPD exacerbations. Chest. 2010;138(4):896-904. Epub 2010/05/04. doi: 10.1378/chest.09-2225. PubMed PMID: 20435659.

72. De Serres G, Lampron N, La Forge J, Rouleau I, Bourbeau J, Weiss K, et al. Importance of viral and bacterial infections in chronic obstructive pulmonary disease exacerbations. Journal of clinical virology : the official publication of the Pan American Society for Clinical Virology. 2009;46(2):129-33. Epub 2009/08/12. doi: 10.1016/j.jcv.2009.07.010. PubMed PMID: 19665425.

73. Ljungman P, Ward KN, Crooks BN, Parker A, Martino R, Shaw PJ, et al. Respiratory virus infections after stem cell transplantation: a prospective study from the Infectious Diseases Working Party of the European Group for Blood and Marrow Transplantation. Bone marrow transplantation. 2001;28(5):479-84. Epub 2001/10/11. doi: 10.1038/sj.bmt.1703139. PubMed PMID: 11593321.

74. Carrat F, Leruez-Ville M, Tonnellier M, Baudel JL, Deshayes J, Meyer P, et al. A virologic survey of patients admitted to a critical care unit for acute cardiorespiratory failure. Intensive Care Medicine. 2006;32(1):156-9. Epub 2005/12/06. doi: 10.1007/s00134-005-2861-4. PubMed PMID: 16328219.

75. Dimopoulos G, Lerikou M, Tsiodras S, Chranioti A, Perros E, Anagnostopoulou U, et al. Viral epidemiology of acute exacerbations of chronic obstructive pulmonary disease. Pulmonary Pharmacology & Therapeutics. 2012;25(1):12-8. Epub 2011/10/11. doi: 10.1016/j.pupt.2011.08.004. PubMed PMID: 21983132.

76. Ko FW, Ip M, Chan PK, Chan MC, To KW, Ng SS, et al. Viral etiology of acute exacerbations of COPD in Hong Kong. Chest. 2007;132(3):900-8. Epub 2007/06/19. doi: 10.1378/chest.07-0530. PubMed PMID: 17573516.

77. Hosseini SS, Ghasemian E, Jamaati H, Tabaraie B, Amini Z, Cox K. Association between respiratory viruses and exacerbation of COPD: a case-control study. Infectious diseases (London, England). 2015;47(8):523-9. Epub 2015/03/25. doi: 10.3109/23744235.2015.1022873. PubMed PMID: 25800059.

78. Martino R, Porras RP, Rabella N, Williams JV, Ramila E, Margall N, et al. Prospective study of the incidence, clinical features, and outcome of symptomatic upper and lower respiratory tract infections by respiratory viruses in adult recipients of hematopoietic stem cell transplants for hematologic malignancies. Biology of blood and marrow transplantation : Journal of the American Society for Blood and Marrow Transplantation. 2005;11(10):781-96. Epub 2005/09/27. doi: 10.1016/j.bbmt.2005.07.007. PubMed PMID: 16182179; PubMed Central PMCID: PMCPMC3347977.

79. Avetisyan G, Mattsson J, Sparrelid E, Ljungman P. Respiratory syncytial virus infection in recipients of allogeneic stem-cell transplantation: a retrospective study of the incidence, clinical features, and outcome. Transplantation. 2009;88(10):1222-6. Epub 2009/11/26. doi: 10.1097/TP.0b013e3181bb477e. PubMed PMID: 19935377.

80. Berntsson E, Blomberg J, Lagergard T, Trollfors B. Etiology of community-acquired pneumonia in patients requiring hospitalization. European Journal of Clinical Microbiology. 1985;4(3):268-72. Epub 1985/06/01. PubMed PMID: 4018066.

81. Nicholson KG, Kent J, Ireland DC. Respiratory viruses and exacerbations of asthma in adults. British Medical Journal (Clinical research ed). 1993;307(6910):982-6. Epub 1993/10/16. PubMed PMID: 8241910; PubMed Central PMCID: PMCPMC1679193.

82. Seemungal T, Harper-Owen R, Bhowmik A, Moric I, Sanderson G, Message S, et al. Respiratory viruses, symptoms, and inflammatory markers in acute exacerbations and stable chronic obstructive pulmonary disease. American Journal of Respiratory and Critical Care Medicine. 2001;164(9):1618-23. Epub 2001/11/24. doi: 10.1164/ajrccm.164.9.2105011. PubMed PMID: 11719299.

83. Falsey AR, Treanor JJ, Betts RF, Walsh EE. Viral respiratory infections in the institutionalized elderly: clinical and epidemiologic findings. Journal of the American Geriatrics Society. 1992;40(2):115-9. Epub 1992/02/01. PubMed PMID: 1740594.

84. Falsey AR, McCann RM, Hall WJ, Tanner MA, Criddle MM, Formica MA, et al. Acute respiratory tract infection in daycare centers for older persons. Journal of the American Geriatrics Society. 1995;43(1):30-6.

85. Whimbey E, Champlin RE, Couch RB, Englund JA, Goodrich JM, Raad I, et al. Community respiratory virus infections among hospitalized adult bone marrow transplant recipients. Clinical infectious diseases : an official publication of the Infectious Diseases Society of America. 1996;22(5):778-82. Epub 1996/05/01. PubMed PMID: 8722930.

86. Whimbey E, Couch RB, Englund JA, Andreeff M, Goodrich JM, Raad, II, et al. Respiratory syncytial virus pneumonia in hospitalized adult patients with leukemia. Clinical infectious diseases : an official publication of the Infectious Diseases Society of America. 1995;21(2):376-9. Epub 1995/08/01. PubMed PMID: 8562747.

87. Palmer SM, Jr., Henshaw NG, Howell DN, Miller SE, Davis RD, Tapson VF. Community respiratory viral infection in adult lung transplant recipients. Chest. 1998;113(4):944-50. Epub 1998/04/29. PubMed PMID: 9554629.

88. Small TN, Casson A, Malak SF, Boulad F, Kiehn TE, Stiles J, et al. Respiratory syncytial virus infection following hematopoietic stem cell transplantation. Bone marrow transplantation. 2002;29(4):321-7. Epub 2002/03/16. doi: 10.1038/sj.bmt.1703365. PubMed PMID: 11896429.

89. Walsh EE, Falsey AR, Hennessey PA. Respiratory syncytial and other virus infections in persons with chronic cardiopulmonary disease. American Journal of Respiratory and Critical Care Medicine. 1999;160(3):791-5. Epub 1999/09/03. doi: 10.1164/ajrccm.160.3.9901004. PubMed PMID: 10471598.

90. Anaissie EJ, Mahfouz TH, Aslan T, Pouli A, Desikan R, Fassas A, et al. The natural history of respiratory syncytial virus infection in cancer and transplant patients: implications for management. Blood. 2004;103(5):1611-7. Epub 2003/10/04. doi: 10.1182/blood-2003-05-1425. PubMed PMID: 14525792.

91. Lo MS, Lee GM, Gunawardane N, Burchett SK, Lachenauer CS, Lehmann LE. The impact of RSV, adenovirus, influenza, and parainfluenza infection in pediatric patients receiving stem cell transplant, solid organ transplant, or cancer chemotherapy. Pediatric Transplantation. 2013;17(2):133-43. Epub 2012/12/12. doi: 10.1111/petr.12022. PubMed PMID: 23228170.

92. Falsey AR, Hennessey PA, Formica MA, Cox C, Walsh EE. Respiratory syncytial virus infection in elderly and high-risk adults. The New England Journal of Medicine. 2005;352(17):1749-59. Epub 2005/04/29. doi: 10.1056/NEJMoa043951. PubMed PMID: 15858184.

93. Peck AJ, Englund JA, Kuypers J, Guthrie KA, Corey L, Morrow R, et al. Respiratory virus infection among hematopoietic cell transplant recipients: evidence for asymptomatic parainfluenza virus infection. Blood. 2007;110(5):1681-8. Epub 2007/05/16. doi: 10.1182/blood-2006-12-060343. PubMed PMID: 17502457; PubMed Central PMCID: PMCPMC1975849.

94. Martinello RA, Esper F, Weibel C, Ferguson D, Landry ML, Kahn JS. Human metapneumovirus and exacerbations of chronic obstructive pulmonary disease. The Journal of Infection. 2006;53(4):248-54. Epub 2006/01/18. doi: 10.1016/j.jinf.2005.11.010. PubMed PMID: 16412516.

95. Camargo CA, Jr., Ginde AA, Clark S, Cartwright CP, Falsey AR, Niewoehner DE. Viral pathogens in acute exacerbations of chronic obstructive pulmonary disease. Internal and Emergency Medicine. 2008;3(4):355-9. Epub 2008/10/01. doi: 10.1007/s11739-008-0197-0. PubMed PMID: 18825480.
